# Genotyped functional screening of soluble Fab clones enables in-depth analysis of mutation effects

**DOI:** 10.1038/s41598-023-40241-2

**Published:** 2023-08-11

**Authors:** Sami Oksanen, Roope Saarinen, Anttoni Korkiakoski, Urpo Lamminmäki, Tuomas Huovinen

**Affiliations:** Department of Life Sciences, University of Turku, 20520 Turku, Finland

**Keywords:** Drug discovery, Biotechnology, Molecular engineering, Sequencing

## Abstract

Monoclonal antibodies (mAbs) and their fragments are widely used in therapeutics, diagnostics and basic research. Although display methods such as phage display offer high-throughput, affinities of individual antibodies need to be accurately measured in soluble format. We have developed a screening platform capable of providing genotyped functional data from a total of 9216 soluble, individual antigen binding fragment (Fab) clones by employing next-generation sequencing (NGS) with hierarchical indexing. Full-length, paired variable domain sequences (VL–VH) are linked to functional screening data, enabling in-depth analysis of mutation effects. The platform was applied to four phage display-selected scFv/Fab screening projects and one site-saturation VH affinity maturation project. Genotyped functional screening simultaneously enabled the identification of affinity improving mutations in the VH domain of Fab 49A3 recognizing Dengue virus non-structural protein 1 (NS1) serotype 2 and informed on VH residue positions which cannot be changed from wild-type without decreasing the affinity. Genotype-based identification revealed to us the extent of intraclonal signal variance inherent to single point screening data, a phenomenon often overlooked in the field. Moreover, genotyped screening eliminated the redundant selection of identical genotypes for further study and provided a new analysis tool to evaluate the success of phage display selections and remaining clonal diversity in the screened repertoires.

## Introduction

Recombinant antibody engineering is a success story in the field of biotechnology. Monoclonal antibodies (mAbs) and their fragments, such as single-chain variable fragment (scFv) and the fragment antigen-binding (Fab), are widely used in therapeutics, in diagnostics and as tools for basic research^1^. As novel target molecules for these applications are discovered, new or improved binders with high affinity, specificity and desired physiochemical properties are needed.

The smaller size of antibody fragments and the lack of the Fc-domain offers several benefits compared to full-sized IgG, including better tissue penetration^2,3^ and smaller chance for interference in in-vitro diagnostic assays^4^. From the point of view of antibody engineering, the main advantage of the simple structure of antibody fragments is their economical and facile expression in *E. coli*, allowing their use in phage display^5^. Advanced recombinant antibody gene library technologies^6,7^ combined with high-throughput display methods offer an efficient way to enrich binders against a vast variety of targets^8^.

Although the display methods have high throughput, the affinity and specificity of individual binders need to be assessed by screening of large number of individual antibodies in soluble format. Loss of specificity has been reported in some studies after phage displayed antibody fragments were converted into soluble format^9,10^. This could be explained by altered conformation of the scFv after losing the support from pIII protein, which is the most common fusion partner of scFv for display^10^. There are also reports on loss of affinity after direct conversion from scFv to IgG format^11,12^, greatly limiting application areas of the discovered antibodies. Furthermore, unintended multimerization of scFvs leads to stronger binding through avidity effects, which is not desired in panning or soluble screening^13,14^. Fabs, however, are generally considered as monomeric molecules which is the ideal screening format for affinity assays and, although the existence of phage particles with multiple displayed Fabs cannot be ruled out, the risk for enrichment due to avidity effects is lower with Fab than scFv phage libraries^13^. In addition to reliable affinity estimation, soluble screening enables the identification of clones with good expression levels.

Enzyme-linked immunoassays (ELISA) and Sanger sequencing have commonly been used in antibody screening and characterization^15–17^. After primary screening, the most promising binder subset can be taken directly to use or further improved via affinity maturation^18–20^. While ELISA methods can rather easily be scaled up, sequencing the screened samples by Sanger sequencing rapidly becomes uneconomical (fixed sample price, for our laboratory ranging from $\sim$ 4 to 6 € at the time of writing, including DNA purification with Thermo GenJet miniprep Kit) and laborious with increasing sample size. Traditionally, only a handful of screened clones are sequenced before further characterization^15–17^ and often the same enriched clones are sequenced multiple times. In addition, selecting candidate clones based on a single ELISA data point from screening assays leaves the analysis susceptible to bias caused by signal variation, e.g. affected by expression differences. Combining geno- and phenotypes already at the screening phase would expose intraclonal signal variation and avoid unnecessary, repeated expression and sequencing. Using high-throughput sequencing technologies (e.g. Illumina) with nested, quadruple index tagging^21–23^ permits sequencing of thousands of individual samples in a single sequencing run. Because of unique tags, samples can be pooled together for library preparation, greatly decreasing per-sample-price as the number of samples increases.

NGS has already been widely applied to antibody engineering^24^, e.g. to evaluate the quality of synthetic antibody libraries^25^ and bypass the primary screening step by observing the enrichment of antibody sequences between the selection rounds^26^. Insights gained with NGS have also been used to develop novel antibodies with improved stability^27^, selectivity and affinity^28^. While most of the current studies focus on sequencing antibody library pools, NGS has also been employed to sequence individual FACS sorted hybridoma cell antibody clones by PCR-barcoding^29^.

Here, we have developed an automation-ready soluble Fab screening platform, which provides genotype-linked functional binding data from thousands of individual Fab clones. Illumina NGS is used with nested, quadruple indexing to sequence variable domain genes of all, up to 9216 functionally screened clones. The nested barcodes link the sequences to their physical locations on the ELISA screening plates. The outer barcodes indicate the plate number, while the inner barcodes indicate the well coordinates. To fully sequence Fab variable region genes with short read sequencing and maintain VH–VL linkage, both domains are tagged with identical internal (well) indexes and sequenced using paired-end mode (301 + 301 bases from each direction). Our hypothesis was, that by utilizing NGS parallel to functional screening, complete geno-phenotype maps could be constructed from site-directed mutant library screening data. Truly unique clones can be identified from the functional data, which increases the accuracy of screening and avoids selection of repeated genotypes for more detailed characterization. Also, retrospective quality analysis of selections and library preparation is made possible.

## Results

### Indexing strategy and validation

**Figure 1:**
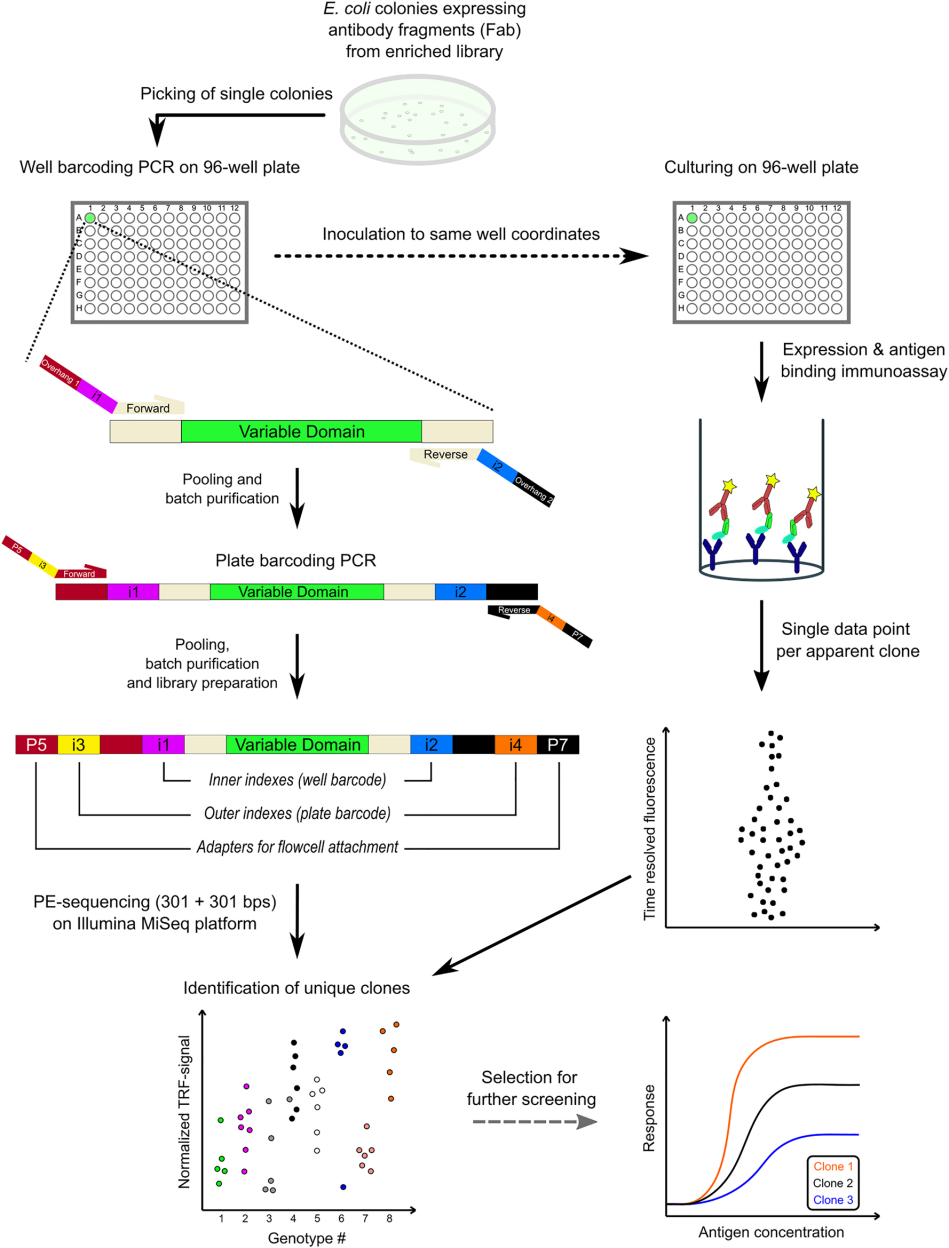
Screening strategy illustrated. Single clones are first inoculated to well-barcoding PCR reaction, and then to corresponding coordinates on expression plate. Variable domains of all clones are amplified with custom well index primers, followed with pooling and purifying all 96 samples in single batch. In plate-barcoding PCR, pooled samples from different plates are tagged with TruSeq index adapters. Samples are again pooled, batch-purified and prepared for sequencing on MiSeq. In parallel (right), functional screening of single Fab clones is carried out. Resulting immunoassay data is then combined with NGS data, truly unique clones identified and best candidates selected for more thorough affinity screening.

To create a robust, cost efficient and automation-ready Fab screening platform, capable of providing genotype-linked functional data from thousands of clones *en masse*, a hierarchical quadruple-index barcoding strategy was developed. Starting from a phage display enriched Fab library, expressed in *E. coli*, single colonies were inoculated from agar plates to a first PCR reaction on 96-well plates, and from there to corresponding well coordinates on expression plates with culture media. In the first PCR step the VH and VL domain genes are amplified in the same reaction with custom primers (Table 1), containing unique index pairs for each well and an overhang for Illumina TruSeq indexing primer hybridization. After the well indexing PCR, the samples from a single plate can be pooled and purified as one sample. In a second PCR step, the plate indexes are added with Illumina TruSeq indexing primers, after which all samples can again be pooled and purified in single batch. After size selection and quantification, the library is ready to be sequenced on an Illumina MiSeq sequencer. In parallel, functional screening of the Fab clones is carried out on 96-well plates, resulting in antigen binding data. The current platform has the capacity to encode a total of 9216 wells by hierarchical indexing. As three positive controls and three empty wells were included on each 96-well plate, 8640 novel Fab clones were sequenced in this study in a single Illumina run. Positive control Fabs selected from the same libraries were included to show if the expression on that plate was successful. The three empty wells also served as negative controls for the well barcoding PCR, and the absence of amplification product was verified by agarose gel electrophoresis of one to three randomly selected plates from each screening project (data not shown). The designed screening strategy is illustrated in Fig. 1. Taking into account the cost of primers, DNA purification, library preparation, quality control and sequencing with MiSeq v3 kit, price per sample/well was 0.35 €. In comparison, Sanger sequencing would have cost approximately 8.5 € per sample, if both VL and VH domains were sequenced separately.

**Table 1:** Custom index primer sequences represented in three pieces in 5$^{\prime }$–3$^{\prime }$ orientation, that together form a single, contiguous primer.

Primer name	TruSeq overhang	Custom index	Hybridization*
Forward 1	TACACGACGCTCTTCCGATCT	CCTAAA	a or b
Forward 2	TACACGACGCTCTTCCGATCT	TGCAGA	a or b
Forward 3	TACACGACGCTCTTCCGATCT	CCATCA	a or b
Forward 4	TACACGACGCTCTTCCGATCT	GTGGTAT	a or b
Forward 5	TACACGACGCTCTTCCGATCT	ACTTTAA	a or b
Forward 6	TACACGACGCTCTTCCGATCT	GAGCAAC	a or b
Forward 7	TACACGACGCTCTTCCGATCT	TGTTGCGT	a or b
Forward 8	TACACGACGCTCTTCCGATCT	ATGTCCGA	a or b
Forward 9	TACACGACGCTCTTCCGATCT	AGGTACGC	a or b
Forward 10	TACACGACGCTCTTCCGATCT	ACAGCCACC	a or b
Forward 11	TACACGACGCTCTTCCGATCT	TGTCTCGCA	a or b
Forward 12	TACACGACGCTCTTCCGATCT	GAGGAGTAA	a or b
Reverse A	CAACGATCGTCGAAATTCGC	CCATCA	c or d
Reverse B	CAACGATCGTCGAAATTCGC	GTTACG	c or d
Reverse C	CAACGATCGTCGAAATTCGC	GTGGTAT	c or d
Reverse D	CAACGATCGTCGAAATTCGC	ACTTTAA	c or d
Reverse E	CAACGATCGTCGAAATTCGC	TGTTGCGT	c or d
Reverse F	CAACGATCGTCGAAATTCGC	ATGTCCGA	c or d
Reverse G	CAACGATCGTCGAAATTCGC	ACAGCCACC	c or d
Reverse H	CAACGATCGTCGAAATTCGC	TGTCTCGCA	c or d

All used indexes have an edit distance $\ge$ 3.

*Separate indexing primers for variable light (a & c) and variable heavy domains (b & d). a = ATTGTTATTACTCGCGGCCCAGC, b = ATCTTCTGCCGACTGCTGCG, c = TAAAAACACTAGGTGCGGCCACAGT, d = TTTAGAAGACGGTGCCAGCGG.

Before application of the strategy to screening projects, the indexing PCR reactions were tested and optimized with control Fab DNA to work in optimal 10 $\upmu$L reaction volumes to be cost-efficient, yet practically feasible for liquid handling. Control Fab sequences were verified with Sanger sequencing to be correct and contained all four indexes and Illumina TruSeq adapter sequences (Fig. S1). In addition, gel electrophoresis analysis showed that all 96 + 96 indexing primer pairs worked with similar amplification efficiency (Fig. S2), allowing simple volumetric pooling of the samples. Furthermore, both VL and VH sequences could be amplified in a single PCR with two pairs of primers. Correct amplification for VH and VL barcoding was again verified with electrophoresis, after PstI digestion of VL amplicon for better separation of VH and VL fragments (Fig. S3).

### Functional screening of soluble Fabs

Antibody Fab fragments against four different antigens (SpyCatcher, DARPin, Dengue virus non-structural protein 1 (NS1) and SARS-CoV-2 nucleocapsid protein (NP)) were screened using the developed platform. Excluding the anti-NS1 library, the Fabs originated from phage display selections from our two synthetic scFv libraries. Selections were started with three to four rounds of phage panning as scFvs, followed by conversion to Fab libraries. Conversion contained a shuffling step for variable light (VL) and variable heavy (VH) pairs, as the domains were first separately amplified from scFv-phage output pools and then cloned with type IIS restriction enzymes or selective RCA into Fab cassettes. After three to four additional rounds of phage display selection, the enriched Fab libraries were cloned with type IIS restriction enzyme to an expression vector, from which the Fabs were expressed as soluble periplasmic proteins with a His-Tag at the C-terminus of the heavy chain. Expression and functional screening with antigen binding immunoassays were conducted on 96-well plates. Antigen concentration for soluble screening was chosen based on assays carried out with control Fabs from earlier studies providing 20% of maximum signal in antigen saturation binding assay. Twenty plates were screened from both anti-SpyCatcher and anti-DARPin libraries. The anti-NP library was split into two sublibraries after the Fab conversion. The first sublibrary, later referred as “NP-1”, was phage display enriched for two rounds to bind the antigen captured by biotinylated Fab N1G1 (clone found during pre-screening of the libraries). The second sublibrary, later referred as “NP-2”, was enriched via phage display for two rounds directly against biotinylated antigen. Eighteen plates from both NP libraries were screened.

Unlike in the other three projects, anti-NS1 Fabs did not undergo phage display selection. The rationale was to broaden the specificity profile of an existing anti-NS1 Fab 49A3 to recognize more uniformly all NS1 serotypes 1–4. To this end, we focused on screening parental anti-NS1 Fab 49A3 variants containing single substitution mutations against Dengue NS serotype 2 antigen, which was recognized with the lowest affinity by the parental Fab compared to the other three serotypes (data not shown). The NS1 screening campaign is analysed in detail in later sections below. For all four screening projects, each screened plate included three control Fab wells (inoculated from colonies similarly to screened clones) and three empty control wells.

**Figure 2:**
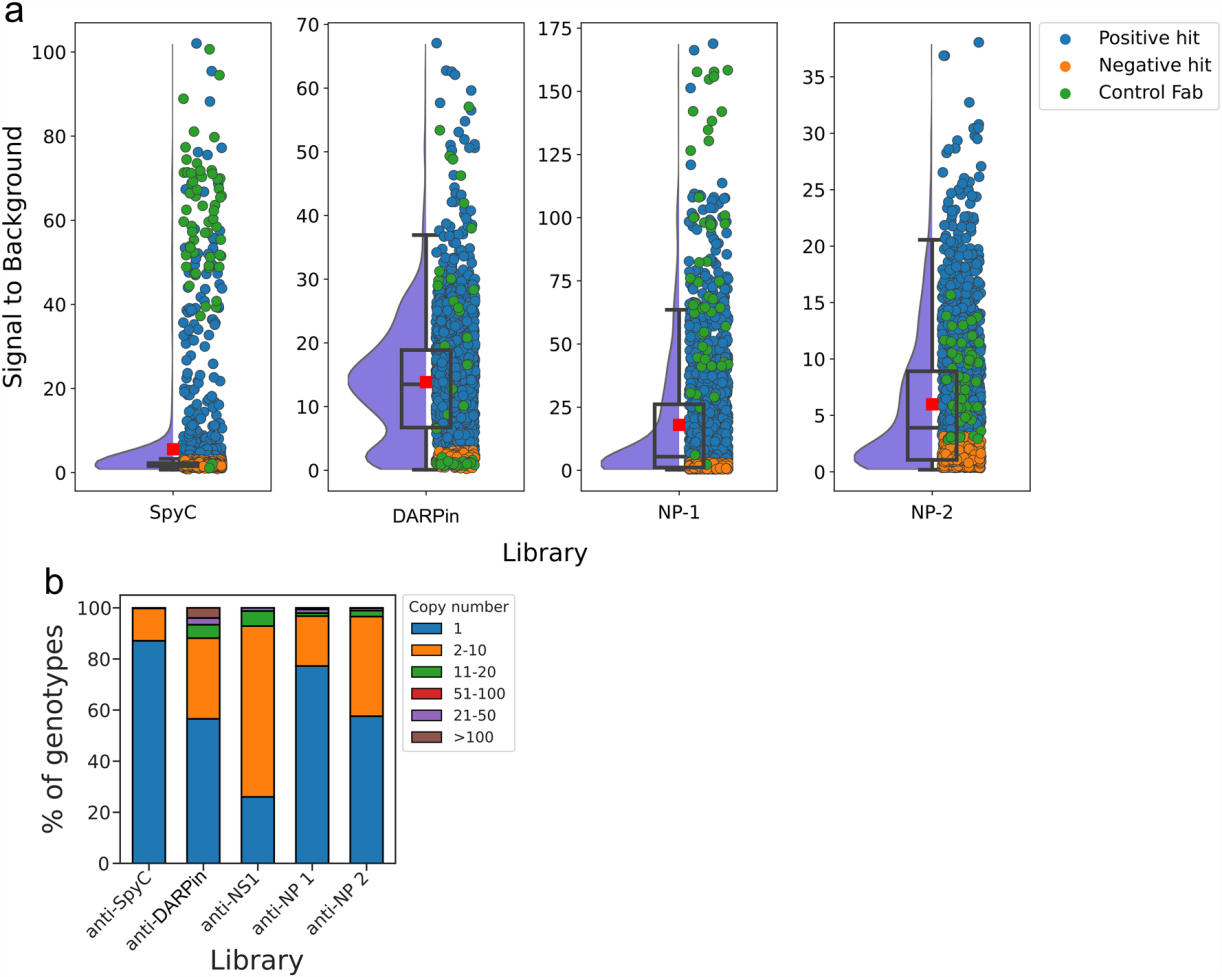
Screening summary of phage display selected genotype-phenotype -screened Fab repertoires. **(a)** Storm cloud plots of functional screening data from antigen binding immunossay for Fab library clones against SpyCatcher, DARPin and SARS-CoV-2 NP, in respective order from left to right. Antigen binding is shown on y-axis as time-resolved fluorescence units divided by empty control well signal. Relative distribution density is illustrated as half violin plot (purple). Median, and quartiles are shown with a boxplot and mean with a red rectangle. Measured data from individual Fab clones are shown with a dot, separating positive hits (blue) with S/B higher than three from negative hits (orange). In addition, control Fab clones are colored with green. (**b**) Prevalence of unique genotypes as copy numbers within each screened library.

In all four screening campaigns, negative control wells yielded uniform, low time-resolved fluorescence (TRF) signal when compared between different plates. The positive Fab control wells showed similar, uniform signal within the same plate in all libraries except in anti-DARPin, where high variance between the three positive controls was observed within the plates. Data normalization was thus done by dividing the TRF signal of screened clones by that of the empty wells (signal to background, S/B). 57% of the screened clones yielded S/B lower than 3, indicating either wells with low Fab expression or poorly performing clones. Positive hit rates with this cutoff were 13, 84, 55 and 54% for anti-SpyCatcher, -DARPin, -NP-1 and -NP-2 libraries, respectively. The functional screening data for each project is illustrated in Fig. 2, and per screened plate in Figs. S4 and S5.

### Analysis of the sequencing data

#### Distribution of reads across the indexed 96-well plates

**Figure 3:**
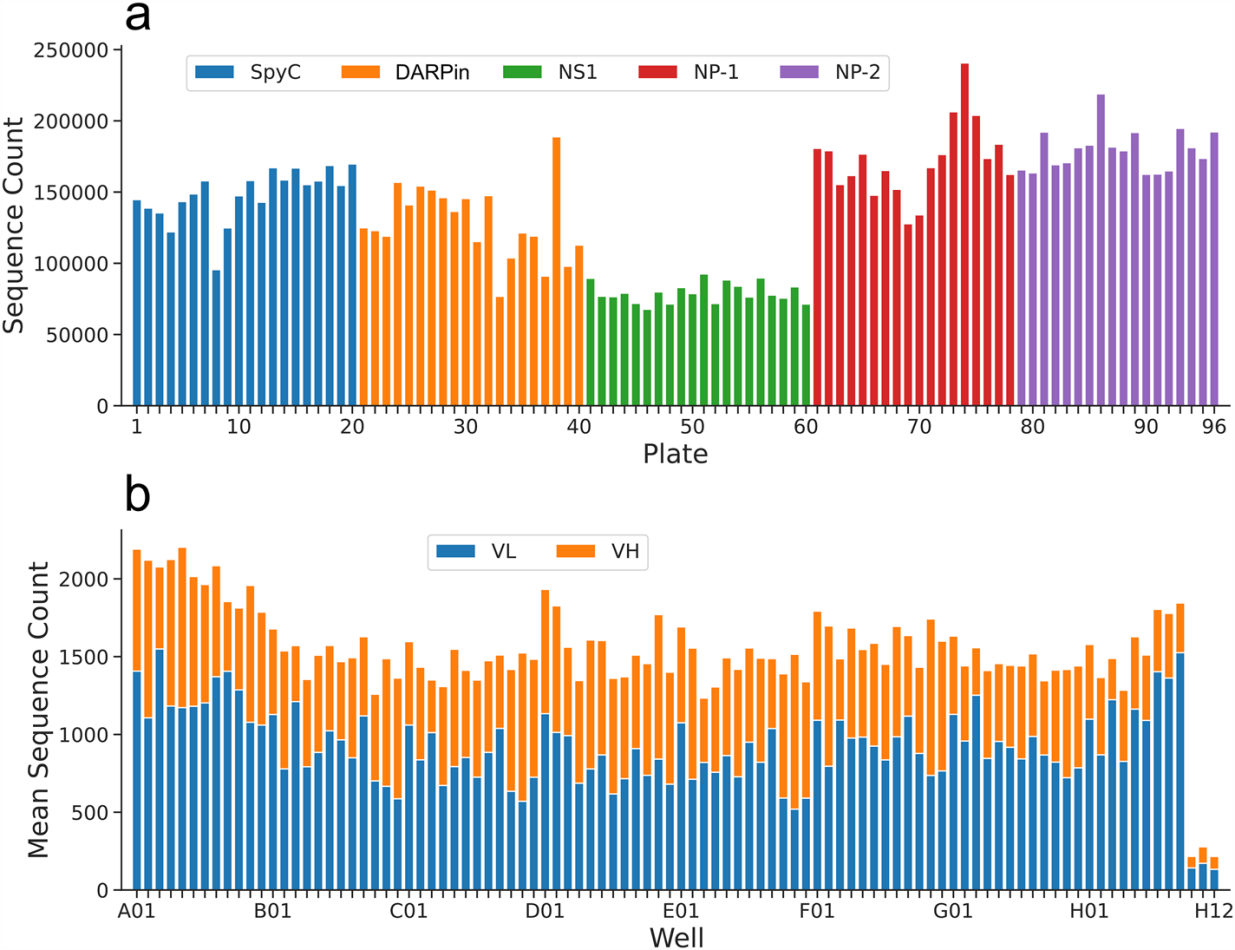
Total sequence count distribution within plate indexes (**a**) and mean sequence count distribution within well indexes (**b**). (**a**) Even distribution of total read counts is observed within the plates. Total sequence counts of anti-NS1 library are slightly lower, as only VH domains were sequenced. SpyC, DARPin, NS1, NP-1 and NP-2 libraries are illustrated with blue, orange, green, red and purple colors, respectively. (**b**) Similarly, balanced mean read count distribution is seen within well indexes after demultiplexing. VL (blue) sequences are moderately more abundant in the data than VH sequences (orange). Very low number of reads origin from wells H10–H12, as they served as empty control wells in screening with no Fab expressing *E. coli* clones in them.

Total of 15 million paired end reads (301 + 301) with average Phred quality score of 32 per read were obtained from sequencing with MiSeq. 89.5 % of the reads were correctly demultiplexed based on TruSeq plate indexes automatically by the Illumina software. The remaining 10.5% of the reads were marked as undetermined, and later identified as PhiX control sequences with BLAST search. This was in par with the spiked amount (10%) of PhiX control. Identified reads were evenly distributed among the plate indexes as shown in Fig. 3a, indicating good input normalization by mass of DNA in library preparation step. Similarly, a balanced read distribution was observed on individual plates between well indexes (Fig. 3b). Reads were also obtained with control indexes (H10–H12), that should not have DNA template. There were on average 229 reads per empty well index compared to average of 1540 reads with indexes referring to wells containing plasmid-bearing bacteria, respectively. Reads of VL domain were moderately more abundant than of VH. Ninety eight percent of the paired reads were successfully merged, providing full length VL and VH domains with flanking well indexes, increasing the average per read Phred quality score to 37. After demultiplexing of custom well indexes and trimming of adapters, 92% of reads qualified for VL and VH sequence analysis. Extraction of VH and VL sequences was done using UNIX commands with custom regular expression patterns. Aligning reads to known control Fab sequences further verified the success of dual index sequencing. Sequencing statistics are shown in more detail in Table 2a.

#### Optimization of the plate indexing PCR reduces the number of sequence chimeras

To our surprise, several different sequences were observed containing the same well ID after collapsing the reads with FASTX-Toolkit. For the majority of clones, we observed distinct single VH and VL sequence with a high abundance, which we presumed to be the correct sequence for that specific index pair. However, multiple samples with no clear main sequence remained (20% of samples had less than a twofold difference between the most and the second most abundant sequence read counts). The contaminating background sequences, also consisting of full-length VH/VL sequences, exhibited library-specific patterns. Identical sequence motifs were identified across different well and plate indexes within the same library, but not across sequenced repertoires of Fabs against different antigens. Completely new amplicons of correct size, not identified as main VH or VL sequence of any clone, were also discovered, further supporting the theory of PCR chimera formation. The chimeras can emerge during the plate barcoding PCR, in which all 93 DNA clones were present as templates in the same reaction and due to high sequence similarity can prime each other and generate novel variants. We further optimized the PCR by changing polymerase to Q5 from Phusion and lowering the number of cycles to eight from original 20. After new sequencing, a clear improvement in sequence clarity was observed. When excluding the empty well indexes from the analyses, an average 1.3 and 2.5-fold improvement was observed in top (main) sequence count to total count and top sequence count to second most abundant sequence count within the same index, respectively (ratios and fold changes shown in Figs. S6, S7).

#### Filtering method improves the assignment of sequence ID to well

**Figure 4:**
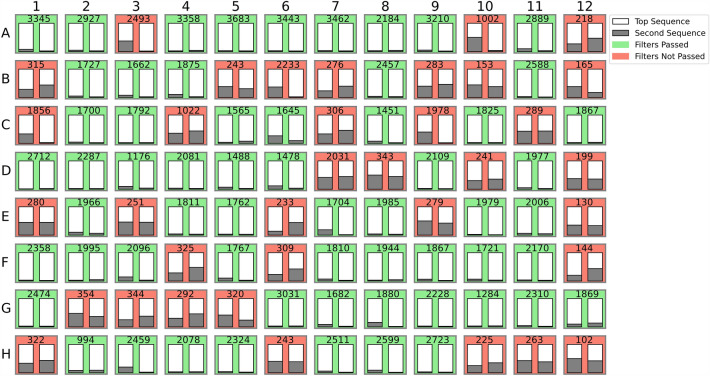
Filtering applied on anti-SARS-CoV-2 NP screening plate S079. Each subplot represents a well on a screening plate, shown in x- and y-axes. The filters passed clones are shown in green and non-passing in red color. Number on top of the subplot shows total number of sequencing reads obtained from that well indexes. VL (left) and VH (right) sequence clarity is shown as bars, showing the proportion of most abundant sequence count (white bar) of that well in comparison to the second most abundant sequence count (gray bar).

After optimized barcoding PCR, some level of background sequences still persisted across all index positions. The empty wells exhibited significantly lower read counts for each variable domain (ranging from 5 to 677, with a median of 95) compared to the experimental wells (ranging from 4 to 4378, with a median of 720). Furthermore, the ratios of the most abundant sequence counts to the second most abundant sequence were, on average, 31-fold higher in the sample wells than in the empty wells, respectively. To ensure systematic and objective sequence assignment for each well, a filtering method was developed. Analysis of total read counts retrieved from individual wells of a 96-well plate guided us to develop a filtering method based on two criteria: (1) the ratio of the most abundant read to the total number of reads per well is higher than equivalent ratio calculated from empty well controls and (2) the ratio of the most abundant read to the second abundant read is higher than twofold. As the total sequence counts varied slightly among the plate IDs, the first filter was calculated individually for each plate. The twofold-threshold value set for the second filter was regarded as a reliable indicator of correct sequence assignment to a well, based on the Sanger sequencing verified data (not shown). After the two filters, the VH and VL (or only VH for anti-NS1 library) sequence containing clone count was 6378 (69% out of theoretical maximum number of clones, including empty well controls from all 96 screening plates). Number of both filters passing clones per screened library (excluding the three wells that were intentionally left empty for control) were 1691 (91%), 1374 (74%), 1520 (81.7%), 1143 (68%) and 1615 (97%) for anti-SpyC, anti-DARPin, anti-NS1, anti-NP 1 and anti-NP 2 libraries, respectively. As the final filter, sequences that were not recognized as antibody variable sequences by the antibody numbering tool ANARCI were discarded before the selection of clones for EC$_{50}$ screening. This resulted in the exclusion of an additional 126 clones, all of which had stop codons in either one of their translated variable domain sequences. 80.9% of control Fab wells passed all the filters, with the majority of non-passing clones belonging to anti-DARPin project. The filtering statistics are shown in Table 2b. An example of the filtering applied on sequence data from a anti-SARS-CoV-2 NP screening plate number S079 is shown in Fig. 4. All clones on plate S079 passed the ANARCI filter.

To verify the fidelity of sequences obtained through demultiplexing the dual-indexed NGS data and applied filtering methods, the 96-well plate named “RS016” containing anti-SARS Cov-2 NP Fab clones was sequenced by Sanger sequencing and the results compared to the NGS-derived sequences. 130 out of 156 (83.3%) good quality Sanger sequences matched completely with sequences obtained with MiSeq. Among the non-matching Sanger sequences, one sample contained a doublet sequence as a result of picking two overlapping colonies from an agar plate. The rest of the assigned nonmatching sequences determined by NGS were identified to be other Fab sequences from the same library output (19 clones), possibly indicating a minor cross-contamination or human error in colony picking. There was no significant difference between the number of mismatching sequences in VH (n = 16) and VL (n = 14). Although the sample size was limited, the results suggest that the vast majority of the clone sequences are correct. However, it is important to acknowledge that there is still a possibility of erroneous sequence assignment due to manual handling of the plates. Additional quality control was performed by comparing sequences of the internal positive control Fab index positions from each screening plate to known control Fab sequences. The demultiplexed and annotated VL and VH sequences that passed the developed filtering method matched perfectly (100%) with the known control Fab sequences in all libraries.

**Table 2:** Sequencing and filtering statistics.

	Read count* (% of total)	Avg. read length (SD)	Avg. Phred score (SD)
(a) Preprocessing			
Undetermined raw reads	1,581,416 (10.5)	301 (0)	31.4 (6.0)
TruSeq demultiplexed raw reads	13,438,539 (89.5)	301 (0)	32.0 (1.5)
Merged reads	13,146,861 (87.5)	439.3 (19.4)	36.9 (2.0)
Demultiplexed & trimmed reads	12,403,861 (82.6)	338.8 (15.3)	NA**

	VL sequence count	VH sequence count	Complete clones (%)***
(b) Filtering			
Not filtered	7296 (100)	9216 (100)	9216 (100)
Empty filter	5519 (75.6)	7352 (79.8)	6777 (73.5)
Noise filter	5346 (73.3)	6882 (74.7)	6378 (69.2)
ANARCI filter	5225 (71.6)	6867 (74.5)	6252 (67.8)

Empty filter = top-to-total sequence count of the sample compared to the value from empty wells of same plate.

Noise filter = top sequence count compared to second most abundant sequence count of the sample.

ANARCI filter = translated sequences recognized as variable domains by antibody numbering tool ANARCI.

*All read counts are reported as read pairs.

** Custom indexed sequences demultiplexed into FASTA files.

*** Both VL and VH (or VH for anti-NS1 library) sequences retained.

### Genotype identification enables more sophisticated selection of candidate binders

#### Clone selection by functional signal alone results in redundant collection of genotypes

**Figure 5:**
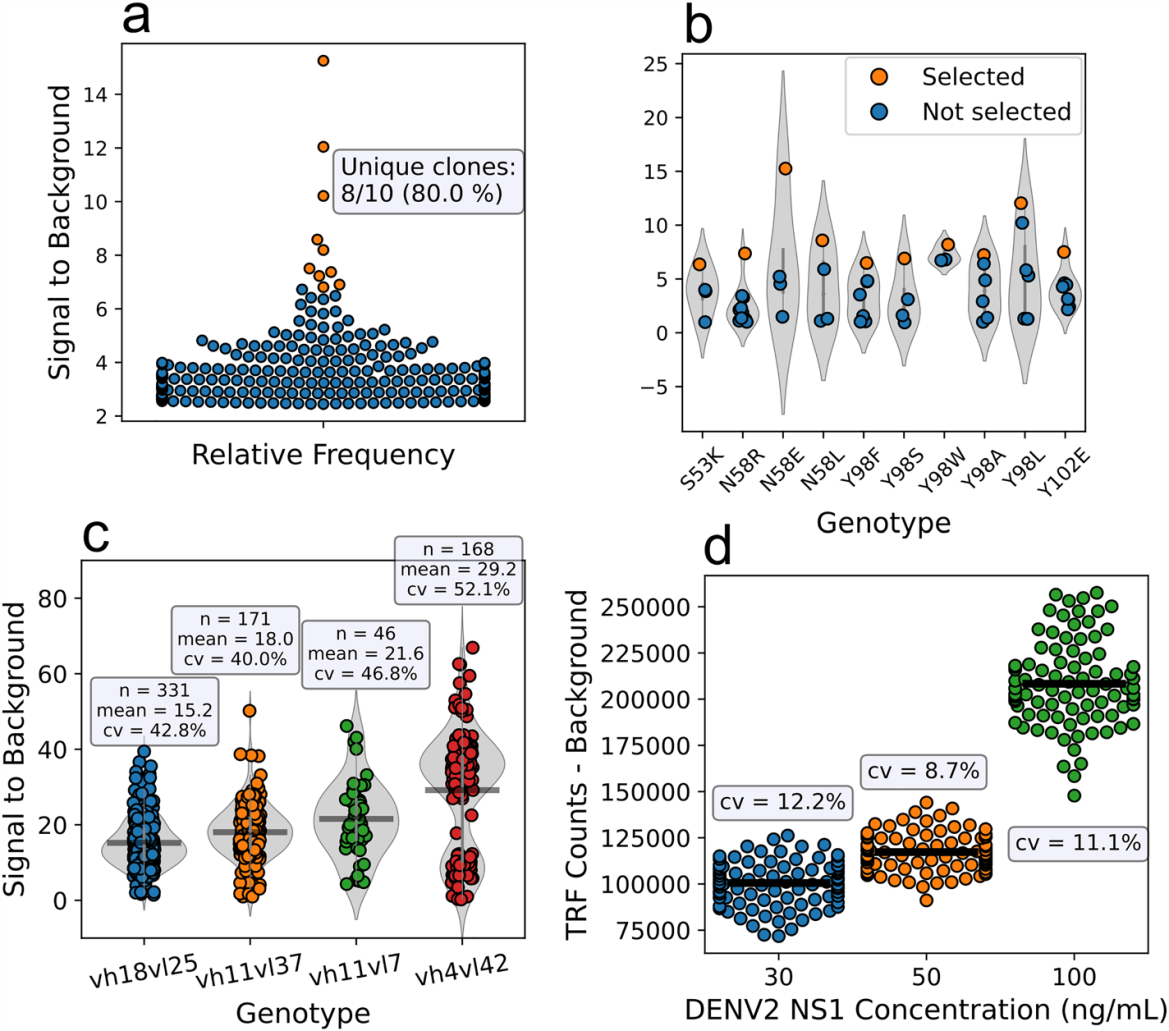
Selection of individual Fab clones for secondary screening. (**a,b**) Clone selection from anti-NS1 Fab library screening using bio-DENV2-NS1 concentration of 50 ng/mL. Gray violin represents the distribution of the individual data points. (**a**) 10 clones with highest signal to background were selected without knowing their genotypes, resulting in selecting two genotypes, Y98W and Y98L, twice. 300 clones with highest S/B are shown in the plot. (**b**) 10 unique genotypes with highest signal to background were selected, avoiding repeated selection and showing variation within each clones binding signal from TRF assay. (**c**) TRF signal variation of four most abundant genotypes in anti-DARPin library screening. Mean values is shown as horizontal line. (**d**) 96-well mock screening with anti-DENV2 NS1 Fab S055B12, demonstration binding signal variation from immunoassay with antigen concentrations of 30, 50 and 100 ng/mL. Average coefficient of variance was 10.7%.

Genotype identification before selecting individual clones for more thorough secondary screening removes the risk of secondary screening the same clone repeatedly, saving resources and time. By selecting clones based on single point TRF assay signals, repeated genotypes were present among the top 10 clones with highest S/B. Proportions of truly unique clones were 8/10, 1/10, 8/10, 9/10 and 9/10 for libraries anti-SpyC, anti-DARPin, anti-NS1, anti-NP 1 and anti-NP 2, respectively. The number of truly unique clones was even lower when 20 clones were blindly selected, being 15/20, 3/20, 13/20, 12/20 and 14/20 in same order. The selections for anti-NS1 library are shown in Fig. 5a,b and for all five libraries in Figs. S8–S12.

The successful enrichment of binders against antigen of interest can be assessed retrospectively with genotyped screening. Proportions of unique VH+VL combinations were 79, 8, 42 and 41% from total number of filters passing clones for anti-SpyC, anti-DARPin, anti-NP 1 and anti-NP 2 libraries, respectively. In anti-NP 1 and anti-NP 2 libraries the enrichment was clearly visible, as 23 and 42% of the genotypes were represented between 2 and 65 times with medians of 2 and 3, respectively. Based on the large sequence diversity of clones screened from the anti-SpyC library selection, the enrichment was not complete at the screening stage, as only 13% of the genotypes were represented in the data more than once, most of which were discovered 2–10 times. The incompleteness of the phage display selection was further supported by the functional screening data as the screened anti-SpyC repertoires had the lowest hit rate (S/B > 3) among the phage display selected libraries. Among genotypes encountered at least three times in the screening campaign, 66.7% did not bind to the SpyCatcher-antigen (S/B < 3), whereas, of the remaining genotypes, 28.9% showed S/B-ratio both above and below the set threefold threshold ratio, and 4.4 % were positive (S/B > 3) in all instances (6.7% if calculated by S/B > 2). On the contrary, anti-DARPin library was over enriched in relation to the number of clones screened, with only 76 unique genotypes, 43% of which were represented between 2 and 331 times with a standard deviation (SD) of 47. Three genotypes, including one used as control, were present with 170 clones or more. The low number of unique clones in the anti-DARPin Fab repertoire is consistent with the selection scheme, as four rounds of Fab-phage selections were carried out with the anti-DARPin library in contrast to three with anti-SpyC library and one to two with anti-NP libraries. The genotype copy numbers per library are shown in Fig. 2b.

#### Exposing the innate variance of primary screening data

As the method reveals genotypes of all screened clones, the TRF signal data can be grouped based on clone identity. The larger the sample size, the more reliable is the quantitative estimation of the binding strength of the clone compared to peer clones and the more reliable is the estimation of the intra-assay signal variation. By analysing the variance in the magnitude of the assay signal obtained from identical clones, the random error of the screening platform was quantitated. This is shown for anti-NS1 clones in Fig. 5b. The intraclonal variation was highest in the anti-DARPin library, and when the four most abundant genotypes were studied, the coefficient of variance (cv) was found to be between 40 and 52% (Fig. 5c). Being able to see the natural variance allows more accurate assessment of the precision of the screening and therefore, elaborated decisions to be made based on weighted evidence.

To further study the intraclonal screening assay signal variation, a mock screening of a plate containing a single clone (anti-DENV2 NS1 Fab S055B12) and controls was done with the standard protocol. The cv values for background-subtracted binding assay TRF-signals were 12.2, 8.7 and 11.1% for antigen concentrations of 30, 50 and 100 ng/mL, as seen in Fig. 5d. Furthermore, we studied how the variance affects to reliability of clone selection and ranking by conducting a simplified in silico simulation. The simulation was conducted with two sample groups of equal sizes (n = 2–20) that followed a normal distribution with assigned S/B means of three and six, respectively (to imitate the hit-rate limit set earlier and a twofold signal difference). The sample groups were compared with a two-tailed t-test under influence of changing variance (5–50%) by iterating the simulation 1000 times to obtain more accurate estimates of average p-values. As shown in Fig. S13 a sample size of 3 is sufficient to confidentially distinguish a twofold difference (p < 0.05) in functional signal for normally distributed data with variance less than 20%. For higher variance, e.g. 40 and 50% as seen in anti-DARPin screening, each group would need sample sizes of 7 or 11, respectively, to see the difference. For instance, to see a statistically significant difference between mean TRF-signals of anti-DARPin clones vh4vl42 and vh18vl25 (variances 52.1 and 42.8%, respectively) using two-tailed t-test, a minimum sample size of 12 was required, verified by iterations with random sampling from each genotype group.

#### Deep analysis of anti-NS1 Fab single substitution CDRH2 and CDRH3 loop libraries

**Figure 6:**
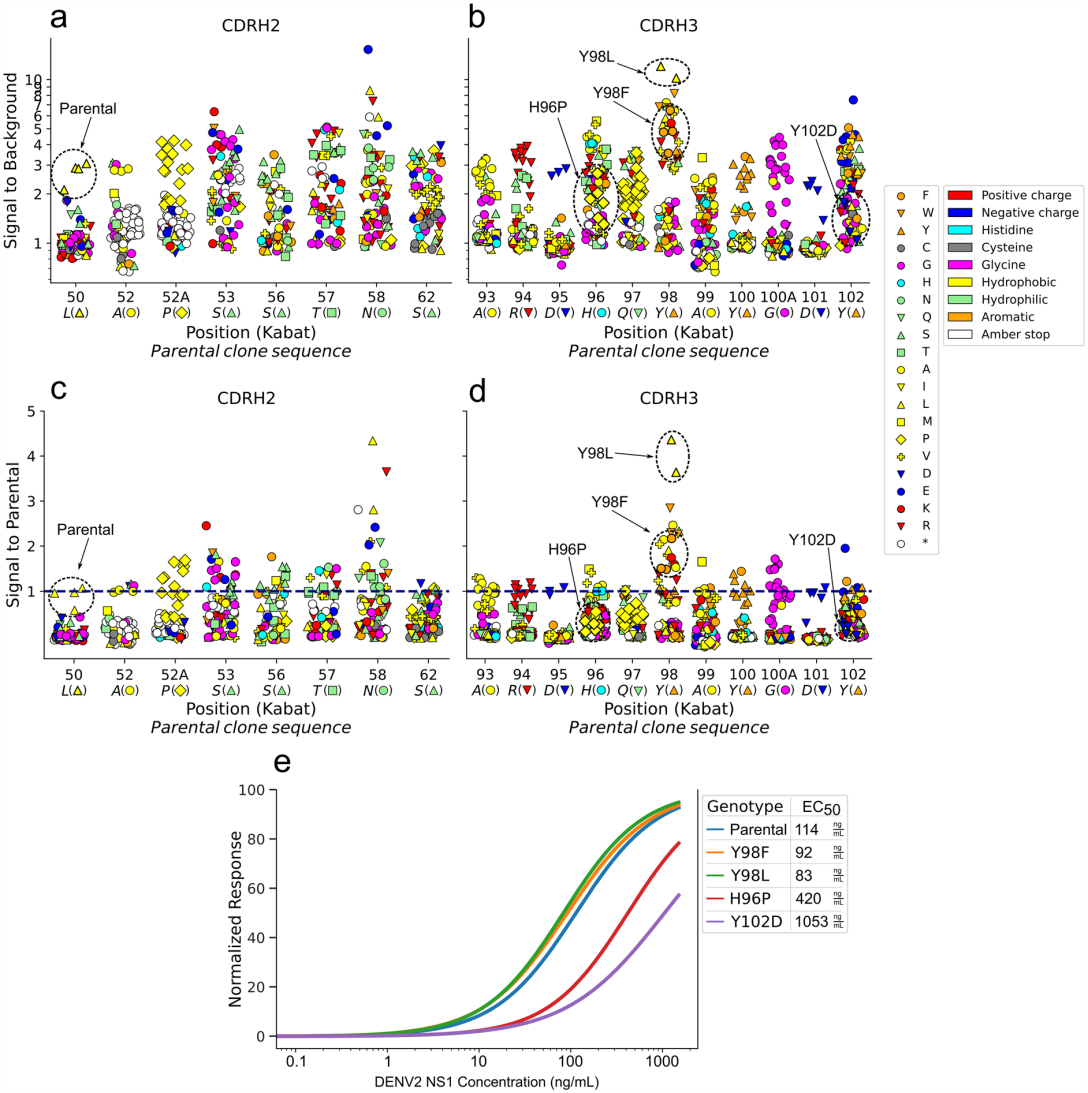
Genotyped functional screening enables in-depth analysis of mutation effects in each targeted position in site-saturation mutagenesis library. (**a–d**) Anti-NS1 functional screening data showing each substitution (symbol) and type of amino acid (color) for sublibraries targeting CDRH2 (**a,c**) and CDRH3 (**b,d**). The measured TRF signal on y-axis is normalized in relation to empty well background (**a,b**) and to parental clone containing wells (**c,d**). A single data point of substitution N58E was excluded from subplot (**c**) (signal to parental of 8.16). Mutation position (Kabat) is shown on x-axis above parental Fab 49A3 genotype (italic). Genotypes presented in subplot **(e)** are annotated with circles, arrows and bolded symbol outlines. The dark blue dashed line in subplots (**c,d**) indicate the parental clone signal level. (**e**) Apparent affinities of selected Fab clones measured with EC$_{50}$ immunoassay.

To investigate whether the anti-NS1 library construction was successful, mutations were studied and unique genotypes counted. The anti-NS1 library consisted of 20 sublibraries, each with a single codon randomized at a specified location in either CDRH2 or CDRH3. One plate per sublibrary was screened with similar controls as before. Each screening plate included three control Fab wells (inoculated from colonies similarly to screened clones) and three empty control wells. Mutations were found from correct positions in all sub-libraries and the coverage of each unique amino acid varied between between 11 and 20. This indicates a successful construction of the sublibraries, with an exception to two targeting positions H52 and H52A (kabat numbering), where the diversity was lower (amber stop and proline were over-represented). The genotyped functional screening data for each sublibrary is shown in Fig. 6a–d.

Apparent affinities of selected Fab clones were measured with EC$_{50}$ immunoassays and compared to the traditional screening data. The clone rank based on S/B value from traditional screening did not perfectly predict the rank based on EC$_{50}$ values in any of the sublibraries (Fig. S14), whether the primary ranking was done for individual apparent clones or identified genotypes as group. When compared to the parental Fab, lower EC$_{50}$ values were observed only for two novel genotypes, Y98L and Y98F, both of which showed a minor 1.37- or 1.24-fold improvement, respectively. Most of the remaining clones showed EC$_{50}$ of two to seven times higher than parental. Nevertheless, the platform enables rapid identification of beneficial mutations from disadvantageous ones in specific position, as shown in Fig. 6. For example, substitution of tyrosine to leusine at position H98 shows improvement in antigen binding over control Fab genotype, which can also be seen as an improvement of apparent affinity (see Fig. S14). Similarly, positions with already optimal residues can be identified (H50, H52A, H93-95, H100 and H100A) from positions that allow substitutions and have room for possible improvements.

## Discussion

In this article we describe a soluble Fab screening platform, capable of providing genotyped functional data from thousands of individual binders by utilizing next-generation sequencing (NGS) with hierarchical indexing. Knowing each clone by their antibody variable domain sequence enables in-depth analysis of mutation effects and reveals the intraclonal variance in primary screening data. This way more educated candidate selections can be done to progress antibody development. Moreover, library quality and success of pre-screening selections can be evaluated retrospectively. Furthermore, selection of clones with shared genotypes can be avoided, saving valuable time and resources. From affinity maturation libraries constructed with site saturation mutagenesis, our platform can reveal beneficial and non-beneficial mutations in each position rapidly from genotyped screening data—something that would not be possible from conventional screening data. Knowing the mutation effects already at the time of primary screening can markedly hasten the antibody engineering process. The favorable substitutions can be studied further either separately or by combining them together for potential additive effect on function, while avoiding disadvantageous mutations.

Positive hit rates (S/B > 3) and distribution of functional screening data differed between the phage-derived screening projects. Despite undergoing 4 + 4 rounds of phage display, it appears that the anti-SpyCatcher library was not fully enriched and could have potentially benefited from an additional round of phage display selection or a reconsideration of the selection strategy. The selection campaign involved various antigens, including chemically biotinylated SpyCatcher, scFv-SpyCatcher, and in vivo biotinylated Spy- and SdyCatcher proteins, aiming to enhance binding towards the catcher rather than the SA-biotin linker. The insufficient enrichment is evidenced by the low number of identical genotypes in the screened set and a relatively low hit rate of 13%. Low hit rate can also be seen in the distribution of the functional signal data (Fig. 2), which is heavily bottom weighted. In the remaining three projects, the hit rates were higher and also the shared genotypes more abundant. In the case of the anti-DARPin library the selection could have been screened after the 3rd round instead of the 4th round of panning, as only a few genotypes were represented in the data, which also had a quite evenly distributed functional signal. NP-1 and NP-2 both come from the same Fab-converted phage library origin, but the functional data between them is very different, with NP-2 having more evenly distributed signals between clones. This was expected, as the last two selection rounds and assay formats were different. NP-2 Fabs were first bound on the wells, followed by addition of biotinylated antigen and detected with Eu-labeled streptavidin, whereas NP-1 Fabs were selected to bind antigen captured by the control Fab N1G1, and the presence of *E. coli*-expressed Fab-APs was detected with europium-labeled anti-alkaline phosphatase antibody.

An anti-NS1 affinity maturation project was selected to test different sample types with our genotyped functional screening platform. Having mutations in targeted, known positions greatly facilitates the data analysis and visualization of mutation effects. In scope of this study, a single plate, i.e. 90 unknown clones, were screened per substitution position. More clones per sub-library would need to be screened in order to obtain a higher replicate number for predicting more accurately the mutation effects considering the inherent variance.

To our knowledge, this is the first report on using NGS to identify thousands of functionally characterized Fabs produced in soluble format in *E. coli*. As mentioned earlier, in antibody engineering NGS has been mostly applied to sequence populations, e.g. enriched phage display pools^25,26,28^ or populations from FACS sorting^27^. Since both heavy and light variable domains contribute to antigen binding, retaining their linkage in sequencing is often crucial. Often compromises are required to link VL and VH, especially when using short-read sequencing technologies such as Illumina. With scFvs, CDRs have been sequenced from single amplicon by reading from CDRL1 to CDRH3^30^, only compromising parts of the framework regions. It is possible to obtain linked VL and VH sequence information of Fab clones with Illumina MiSeq 500 cycle kit by carefully positioning the sequencing primers close to the CDR loops and optimizing the cycle numbers of paired end read lengths (read 1: 270 bases covering VL CDRs + read 2: 230 bases covering CDRH1 and -H2)^31^. There is, however, a risk of losing important information due to loss of base quality towards the end of the MiSeq reads^32^. Another strategy to keep the VH–VL linkage was proposed by Barreto et al. In their study, they used Kunkel mutagenesis to remove intervening framework regions between CDR domains to combine CDRL3–CDRH3 or CDRL3–CDRH1–CDRH2–CDRH3 into single plasmids, where they could be amplified for sequencing on Ion Torrent^33^. The Kunkel approach is still dependent on the sequencing read length which should cover the truncated target size. In our platform, VL and VH are sequenced from different amplicons linked via hierarchical index IDs (Fig. 1), following a similar approach demonstrated in the hybridoma antibody discovery process by Chen et al.^29^ Hierarchical index sequencing, however, does not need to be limited to two amplicons as a custom number of sequencing targets could be amplified and linked with the well indexes for identification.

Due to availability and assured compatibility with Illumina flow cells, we chose to use Illumina proprietary indexing primers from the TruSeq Custom Amplicon kit for plate barcoding. Nevertheless, our custom index primers should also be fully compatible with iTru primers, described in the Adapterama series^22,23,34^, which include all necessary elements for sequencing with universal Illumina sequencing primers. 384 unique index sequences have been developed for both primer pairs in iTru system. Thus, in the future, the throughput of our screening platform could be further increased from current 9216 to up to 14,155,776 (12 $\times$ 8 $\times$ 384 $\times$ 384), if desirable. On average we obtained 1540 reads from indexes referring to wells containing plasmid-bearing bacteria, so a tenfold increase of sample size would still result in 150 or more reads per sample. This is still sufficient for genotype identification assuming there is no change in the ratio of dominant sequence vs. background sequences per well. Undoubtedly, increasing the number of samples would require full automation of the processes. The platform could be fairly easily automated using commercially available liquid handling robots and automated colony pickers or even low-cost, custom made combinations, such as demonstrated by Hartley et al.^35^.

During this proof-of-concept study colony picking to PCR & culturing and pipetting was done manually, and we acknowledge the risk for human error and their effects on the screening data. Major sources of error in manual handling are typically inoculating same well twice or skipping a well either in the culture phase or at adding template material to the PCR plates. In our data we observed both low amount of reads (not passing the “empty well” filter) from wells with with measurable functional signal, indicating low number of bacteria inoculated into PCR reaction, or two distinct high-copy-number clones (not passing the “clarity” filter), which were also verified with Sanger sequencing from the stored culture plate.

The analysis of the hierarchically indexed amplicon sequences was complicated by the presence of unexpected background sequences existing at low copy frequency in each demultiplexed well sequencing sample. Closer inspection of the contaminating reads revealed that identical sequences could be found across different well and plate indexes with the common factor that the plates in question were panned against the same target. The identical background sequences were not found from repertoires selected against other targets. This indicates that they were in fact generated during the second PCR, where 96 well-indexed DNA samples from same plate were amplified as a pool with TruSeq indexing primers. Identical sequences could, naturally, be found from different plate IDs from the same panning campaign, as identical clones were present in parallel screening plates. In addition, completely novel VL and VH sequences were discovered. This is a clear indication of generation of chimeras through PCR recombination, which is a long-known problem when simultaneously amplifying sequences with high similarity^36,37^. We mitigated the chimera formation by lowering cycles from original 20 to 8, as recommended by Lahr et al.^38^ and changing the polymerase from Phusion to Q5. Even though we were able to improve the clarity of the main sequence per index, some level of background remained. Further lowering the amount of template DNA could be beneficial^38^. Individual DNA purification of the well-barcoded PCR amplicons, as demonstrated by Chen et al.^29^, would also result in a lower number of distinct sequences for each well. However, it is important to note that such an approach would significantly increase the cost and workload of projects of this scale. To further eliminate the possibility of chimera formation in the future, outer plate indexes could be added via ligation instead of PCR with high accuracy by using type II restriction enzymes, similarly as was done as first indexing step by Bayona-Vásquez et al.^34^.

Yet another indexing strategy includes increasing the number of unique indexing primers, as was done by Wittmann et al. in 2022^39^. In their study, the gene of interest was amplified from frozen *E. coli* cultures, using low number of cycles and target specific primers with overhangs for index primer annealing. In the second PCR step, still performed for each well separately, the amplicons were further amplified with custom index primers. A total of 96 uniquely indexed forward primers were used to label the well coordinates and 96 uniquely indexed reverse primers to indicate the plate IDs. This allowed pooling of 9216 individual samples and doing further library preparation in single batch, similarly to our approach. After purification and size selection, the sample could be sent to a sequencing service provider, either to be ran among other customer samples with unique Illumina indexes or use the whole flowcell, increasing the read depth. Benefits of this approach include elimination of chimera formation, as PCR reactions only contain single sample, and the possibility to purchase sequencing “slot” from provider instead of using the whole flowcell.

Although our research group has carried out soluble scFv and Fab clone screening with TRF immunoassays routinely for over two decades^40^, the study revealed to us for the first time the true intra-assay variation present in 96-well expressed antibody arrays. The intraclonal variation in the assay signal is most likely due to varying expression levels of Fab clones. As all clones are unique, their specific growth rates can differ from each other, resulting in non-uniform growth phase at induction, which in turn results in variating amounts of Fab expressed. Furthermore, the expressed Fabs may burden the host cell metabolism depending on the sequence details leading to varying toxic effects and Fab yields^41^. Rather high variance was also observed in controlled experiment with same Fab clone, where the coefficient of variance of antigen binding immunoassay TRF signal was around 11%. If the magnitude of variance measured in the control experiment would be generalizable to all screening experiments, at least three biological replicates would be needed to observe twofold difference in functional immunoassay signal with high confidence, as shown with our simulated data. For libraries with low enrichment, definite differences between genotypes cannot be assessed. In addition to growth rate, multiple different persons performing the expressions and functional screening can also affect the differences of data variance.

In conclusion, we have developed a novel soluble Fab screening platform, yielding genotype-linked functional binding data from thousands of binders *en masse*, while retaining the VH–VL linkage. The platform was validated with four screening projects, showing its versatility to aid in candidate selection based on variance analysis and the stage of enrichment achieved in panning experiments. Though our data is sufficient to demonstrate and validate the platform, automation is unquestionably required for true high-throughput screening and better quality screening data. Furthermore, accuracy of the platform could be improved by starting the indexing from *E. coli* culture plate instead of moving each single colony from one well into another, in addition to the chimera mitigation strategies discussed above.

## Methods

### Bacterial strains, vectors and cloning

*E. coli* XL-1 Blue (recA1 endA1 gryA96 thi-1 hsdR17 supE44 relA1 lac [F’ proAB lacI q Z$\Delta$M15 Tn10 (Tet r)]), originally purchased from Stratagene (USA), was used for all phage display selections and Fab expression in screening. pEB32x^6^ phagemid vector (later “display vector”) was used in phage display. pLK06H^6^ and pAK400^42^ vectors were used to express Fabs in soluble format for screening. pUC19 vector (New England Biolabs, USA) was used for Sanger sequencing. For cloning, restriction enzymes SfiI, LguI, HindIII and BamHI and T4 DNA ligase were used according to the manufacturers recommendations, all purchased from Thermo Fisher Scientific, USA. Transformation to *E. coli* was done with electroporation using Gene Pulser Xcell (Bio-Rad, USA) at 1250 V, 25 $\upmu$F, 200 Ohm, with 1 mm Gene Pulse Cuvettes (Bio-Rad, USA). After electroporation, the cells were allowed to recover in 1 mL of SOC media (20 g/L Tryptone, 5 g/L Yeast extract, 0.5 g/L NaCl, 2.5 mM KCl, 10 $\upmu$M MgCl_2_, 3.6 g/L D-Glucose, pH 7.0) for 1 h at 37$^\circ$C with shaking at 300 rpm, followed with inoculation to agar plates with 1 % glucose and suitable antibiotics (10 $\upmu$g/mL tetracycline and 25 $\upmu$g/mL chloramphenicol for pAK400 vectors or 100 $\upmu$g/mL ampicillin for pLK06H). All used antibiotics were purchased from Sigma Aldrich (USA).

### Antibody library construction and selection

Four separate Fab screening projects were conducted to demonstrate the performance of the developed platform. Three of the libraries, targeting SpyCatcher^43^ (later “anti-SpyC”), DARPins^44^ (“anti-DARPin”) or SARS-CoV 2 nucleocapsid protein (consisting of two sublibraries, “anti-NP 1” and “anti-NP 2”) were enriched from our synthetic antibody phage display libraries ScFvM and ScFvP^6^. Briefly, scFvs cloned into display vector were selected utilizing magnetic beads, in first round by saturating the bead surface antigen of interest, and in remaining two to three rounds with antigen in solution mixed phages and recovered with magnetic beads. After scFv selections, conversion to Fabs was done either with selective RCA^45^ (anti-SpyC) or type II restriction enzyme based method, similarly as published earlier^46^, followed with cloning back to display vector. Three to four panning rounds were again conducted in Fab format, similarly to before. After panning, libraries were cloned into expression vectors (pLK06H for anti-NP1 and pAK400 for the rest). Anti-NS1 library was an affinity maturation project constructed with site-saturation mutagenesis (illustrated in Fig. S15). Briefly, anti-NS1 Fab 49A3 (discovered at Department of Life Technologies, University of Turku) DNA was used as template to generate total of 20 sub libraries, each with single codon randomized in CDRH2 (9 sub-libraries) or CDRH3 (11 sub-libraries) using NNK primers ordered from Sigma Aldrich, USA. Generated libraries were directly cloned into pAK400 expression vector for screening. More detailed information about phage display selections, Fab conversions and constructions, including control Fab sequences, can be found in supplementary materials.

### Development and validation of quadruple indexing strategy

Minipreps, gel extractions and PCR purifications were done with GeneJet kits (Thermo Scientific, USA) according to the manufacturers recommendations, unless stated otherwise. Sanger sequencing was bought as service from Macrogen Inc. (South Korea). Two sets (for VH and VL) of 12 forward and 8 reverse well barcoding index primers, containing variable domain hybridization, custom indexes (with minimum edit distance of 3) and Illumina TruSeq custom index adapter hybridization sequences were designed and ordered from Sigma Aldrich (USA) (shown in Table 1). TruSeq Custom Amplicon kit primers (Illumina inc., USA) were used as outer indexes for plate barcoding.

Initial testing of the well index primer pairs was done using 1 ng of purified plasmid DNA of known anti-microcystin Fab 226A2 (discovered at Department of Life Technologies, University of Turku) as a template to PCR amplify VH and VL in 20 $\upmu$L reaction volumes. Later the reaction volume was lowered to 10 $\upmu$L, and simultaneous amplification of the two variable domains was assessed from both purified plasmid DNA and single, Fab expressing XL1 colony as a template. Amplification was assessed with 1% agarose gel electrophoresis. Well barcoding PCR reaction consisted of 0.025 U/$\upmu$L FirePol DNA polymerase (Solis Biodyne, Estonia), 1$\times$ Buffer BD, 1.5 mM MgCl$_2$, 25 $\upmu$M dNTP and 0.2 $\upmu$M primers. Thermocycling was 35 cycles of 95 $^\circ$C (30 s), 56 $^\circ$C (60 s) and 72 $^\circ$C (60 s) with a 95 $^\circ$C (15 min) initial denaturing at the beginning and 72 $^\circ$C (4 min) final elongation step at the end. Following plate barcoding PCR reaction consisted of 0.02 U/$\upmu$L Q5 High-Fidelity DNA Polymerase (New England Biolabs, USA), 1$\times$ Q5 Reaction Buffer, 200 $\upmu$M dNTP, and 0.5 $\upmu$M primers and thermal cycling was 8 cycles of 98 $^\circ$C (10 s), 67 $^\circ$C (15 s) and 72 $^\circ$C (30 s) with a 98 $^\circ$C (3 min) initial denaturing at the beginning and 72 $^\circ$C (4 min) final elongation step at the end.

In addition to electrophoresis, the fidelity of the quadruple indexed variable sequences of Fab 226A2 were Sanger sequenced. First, HindIII and BamHI restriction sites were added with P5 and P7 hybridizing primers (TH260_BamHI-P5: 5$^{\prime }$-cggggatccAATGATACGGCGACCACCGAG-3$^{\prime }$ and TH261_HindIII-P7: 5$^{\prime }$-tgcaagcttCAAGCAGAAGACGGCATACGAGAT-3$^{\prime }$) with similar reaction mix for Q5 High-Fidelity DNA Polymerase as before and thermal cycling of 25 cycles of 98 $^\circ$C (10 s), 69 $^\circ$C (30 s) and 72 $^\circ$C (30 s) with a 98 $^\circ$C (3 min) initial denaturing at the beginning and 72 $^\circ$C (4 min) final elongation step at the end. Amplicons were cloned into pUC19 vector by digesting them with BamHI and HindIII, followed with gel extraction and finally ligation using T4 DNA ligase. Constructs were transformed into XL1 by electroporation, recovered and plated as described earlier. Next morning four colonies from both VH and VL plates were inoculated into 5 mL of SB (30 g/L Tryptone, 20 g/L Yeast extract, 10 g/L MOPS), grown o/n, used for minipreps and DNA sent to sequencing with primer LMB: 5$^{\prime }$-ATGTGCTGCAAGGCGATTAAG-3$^{\prime }$.

### Functional screening of soluble Fabs

For soluble Fab expression, single colonies of XL1 were inoculated from agar plate first to well barcoding PCR well containing 10 $\upmu$L of reaction mix, and from there to primary culture to corresponding well on 96-well plate containing 200 $\upmu$L SB media (30 g/L Tryptone, 20 g/L Yeast extract, 10 g/L MOPS) supplemented with 1 % D-glucose and suitable antibiotics, followed with overnight incubation at 37 $^\circ$C, 900 rpm. After incubation cells were inoculated to expression culture, with four dips using 96-well replicator, into fresh SB supplemented with 0.05% D-glucose and antibiotics and incubated at 37 $^\circ$C with standard shaking for 4 h. Original culture plates were stored at − 70 $^\circ$C after adding glycerol to final concentration of 16 %. Expression was induced by addition of IPTG to final concentration of 200 $\upmu$M, and lowering temperature to 26 $^\circ$C for 16 h. Cells were pelleted by centrifugation at 4 $^\circ$C, 3200$\times$*g* for 30 min. Pellet was resuspended in lysis buffer (1 mg/mL lysozyme from chicken egg white L6876 (Sigma Aldrich, United Kingdom), 0.025 U/$\upmu$L Pierce Universal Nuclease (Thermo Fisher Scientific, USA) in PBS (20 mM sodium phosphate, 300 mM sodium chloride, pH 7.4), followed by 30 min incubation in room temperature in slow agitation, and finally freezing at − 70 $^\circ$C. Lysate was clarified by centrifugation before the immunoassay.

Binding to target antigen was measured with time-resolved fluorescence (TRF) based immunoassays. Antigen concentrations representing EC$_{20}$ values of the control Fabs were used. The clones named S001E03 and N1G1 were used as positive controls for anti-SpyC and anti-NP1 & 2 screening, respectively. They were discovered by pre-screening a single plate (93 clones + 3 empty controls) and selected based on high TRF signals, indicating specific antigen binding and adequate expression levels. Fabs 49A3 (the parental clone for the anti-NS1 project) and 248G12 (the positive control for anti-DARPin screening) were discovered in earlier projects at the Department of Life Technologies, University of Turku, and shown to have good expression levels and antigen binding (not published). The control Fab sequences, with exception to 49A3, are listed in Table S3. The immunoassays were done either on streptavidin (Kaivogen Oy, Finland) or Goat anti-Human (Fab specific, later referred as “GAH”) IgG (Sigma Aldrich, USA) coated plates. GAH plates were prepared by passive coating on C12 MaxiSorp plates (Thermo Fisher Scientific, USA), described in detail in supplementary materials. All dilutions for immunassays were done using Assay Buffer Red and washes using Kaivogen Wash buffer, both purchased from Kaivogen. Delfia Plate Wash (Wallac, Turku Finland) instrument was used for plate washing. All incubations were done at room temperature with slow shaking. Immunoassay plates were read with Victor 1420 Multilabel Counter (Wallac).

For anti-SpyCatcher Fabs, 100 $\upmu$L 1:5 dilution of clarified lysate was added per well on GAH plate and incubated for 1 h, followed with 2 washes. 100 $\upmu$L of 62.35 nM N1-Europium labelled SpyCatcher (produced and labeled at Department of Life Technologies, University of Turku) was added and incubated for 1 h, followed with 4 washes and addition of 200 $\upmu$L Delfia enhancement solution (Kaivogen). After 10 min incubation, TRF was measured Victor.

Similarily for anti-DARPin Fabs, 100 $\upmu$L 1:5 dilution of clarified lysate was added per well on GAH plate and incubated for 1 hour, followed with 2 washes. 100 $\upmu$L of 62.7 nM of biotinylated BiLib DARPin-SpyC (produced and labeled at Department of Life Technologies, University of Turku, see Supplementary Materials) was added and incubated for 1 h, followed with 2 washes and addition of 100 $\upmu$L of N1-Europium labeled streptavidin (later SA-Eu, purchased from BioSpa, Italy and labeled at Department of Life Technologies, University of Turku). After 20 min incubation, wells were washed twice and 200 $\upmu$L of Delfia enhancement solution (Kaivogen) was added, followed with 10 min incubation and TRF measurement with Victor.

For anti-NS1 Fab screening, biotinylated Dengue Virus 2 Non-strutural protein (bio-NS1) was used as antigen (purchased from The Native Antigen Company, UK and biotinylated with EZ-Link NHS-PE$G_4$-Biotin with 12$\times$ molar excess of biotin, according to the manufacturers protocol). Immunoassay was done the same as for anti-DARPin Fabs, with concentration of bio-NS1 being 50 ng/mL. Anti-NP-2 screening was also done on GAH plates with similar protocol, however with shorter incubation times for lysate and antigen (30 min). SARS-CoV-2 Nucleocapsid Protein with GST-tag (kindly provided us by Prof. Julkunen, Institute of Biomedicine, University of Turku, see supplementary materials) was biotinylated in-house similarily to bio-NS1 and used as antigen for screening at concentration of 10 nM. Best candidate Fab, RS016D01, was chosen based on preliminary screening data analysis, was expressed same as described earlier and purified with NiNTA and size exclusion chromatography. RS016 was biotinylated similarily to before and used as capture Fab for anti-NP-1 screening.

In contrast to other screening campaigns, anti-NP-1 screening was concluded on yellow streptavidin plates (Kaivogen Oy, Finland). 60 $\upmu$L of 30 nM bio-RS016 Fab was added to prewashed wells and incubated for 30 min, followed by 4$\times$ wash and addition of 60 $\upmu$L of 10 nM N-GST antigen. After another 30 min incubation and 2 washes, 1:5 dilution of clarified lysate was added per well and incubated 30 min, followed with 2 washes. 60 $\upmu$L of N1-Eu-labeled anti-alkaline phosphatase (AP) polyclonal antibody (anti-AP pAb) (LifeSpan Biosciences, Inc., USA, labeled at Department of Biotechnology, University of Turku^15^) at concentration of 125 ng/mL was added per well, incubated of 30 min and washed 2 times. Enhancement and measurement was done same as above.

### Hierarchical indexing and MiSeq library preparation

In parallel to functional screening of the Fabs, two indexing PCR reactions were done, targeting Fab variable domains. Single bacterial colony from agar plate was picked into 10 $\upmu$L well barcoding PCR reaction mix, prepared on 96-well Armadillo PCR plates (Thermo Fisher Scientific, USA), and from there to expression as described in previous subsection. Well barcoding PCR consisted of 0.025 U/$\upmu$L FirePol DNA polymerase (Solis Biodyne, Estonia), 1$\times$ Buffer BD, 1.5 mM MgCl$_2$, 25 $\upmu$M dNTP and 0.2 $\upmu$M custom indexing primers. Thermocycling was 35 cycles of 95 $^\circ$C (30 s), 56 $^\circ$C (60 s) and 72 $^\circ$C (60 s) with a 95 $^\circ$C (15 min) initial denaturing at the beginning and 72 $^\circ$C (4 min) final elongation step at the end. 5 $\upmu$L from each well were pooled and batch purified using GeneJet PCR purification kit. Following plate barcoding PCR reaction was also concluded on 96-well Armadillo plates at 20 $\upmu$L volume. Original PCR consisted of 1 ng of template DNA, 0.02 U/$\upmu$L Q5 High-Fidelity DNA Polymerase (Thermo Scientific, USA), 1 $\times$ X Phusion^TM^ HF Buffer, 200 $\upmu$M dNTP, and 0.5 $\upmu$M primers with thermal cycling of 20 cycles of 98 $^\circ$C (10 s), 72 $^\circ$C (40 s) with a 98 $^\circ$C (3 min) initial denaturing at the beginning and 72 $^\circ$C (4 min) final elongation step at the end. After discovery of chimera formation, the PCR reaction was optimized as follows; 2 ng of template DNA, 0.02 U/$\upmu$L Q5 High-Fidelity DNA Polymerase (New England Biolabs, USA), 1 x Q5 Reaction Buffer, 200 $\upmu$M dNTP, and 0.5 $\upmu$M primers with thermal cycling of 8 cycles of 98 $^\circ$C (10 s), 67 $^\circ$C (15 s) and 72 $^\circ$C (30 s) with a 98 $^\circ$C (3 min) initial denaturing at the beginning and 72 $^\circ$C (4 min) final elongation step at the end. 15 $\upmu$L from each sample within the same libraries were pooled and purified in batch, similarly to above. Remaining PCR product was used as quality control and used for electrophoresis analysis by running on 1% agarose gel at 70 V for 90 min.

For library size selection and per-library-per-domain input normalization, the fully indexed VH and VL domain genes were separated with 2% agarose gel electrophoresis, ran at 70 V for 2 h, followed by DNA extraction with GeneJet gel extraction kit. All resulting samples were pooled together in equal molar ratios. Sequencing was bought as service from Finnish Functional Genomic Center (Turku, Finland), where library quality was first assessed using 2100 Bioanalyzer with High Sensitivity DNA Assay kit (Agilent, USA) and quantified using Qubit (Thermo Fisher Scientific, USA). 9 pM library supplemented with 10% PhiX Sequencing Control V3 (Illumina, USA) was sequenced on MiSeq using MiSeq Reagent Kit v3 (600-cycle) (Illumina).

### Sanger verification of NGS results

After sequencing of the five Fab library clones, the fidelity of the platform was further verified by Sanger sequencing one of the screened 96-well plates (plate RS016 from anti-NP-2). Cells from glycerol prep plate were inoculated to fresh SB on 96-well plates and cultured o/n same as before. Next morning, 1 $\upmu$L of cells was used in PCR reaction, similar to well barcoding, to amplify the Fab genes using primers ULa03_20: TCCGGCTCGTATGTTGTGTGG and SAk_06: CGAGAAAGGAAGGGAAGAAAGCGAAAGGAG. Thermal cycling was 30 cycles of 95 $^\circ$C (30 s), 65 $^\circ$C (30 s) and 72 $^\circ$C (2 min) with a 95 $^\circ$C (3 min) initial denaturing at the beginning and 72 $^\circ$C (4 min) final elongation step at the end. DNA was purified using PureLink Pro 96 PCR purification kit (Invitrogen, USA) according to manufacturers protocol and sent to sequencing with primers WO375: TCACACAGGAAACAGCTATGAC or HS003: ATCTTCTGCCGACTGCTGCG. Good quality VH and VL sanger sequences were compared to the most abundant sequences in each index position from NGS (exact matching).

### Data analysis

Quality of FASTQ files after each step was analysed using FASTQC^47^ v0.11.9 in conjunction with MultiQC^48^ v1.11. Read pairs were merged and prefiltered using PEAR^49^ v0.9.11 with parameters -v 25 -m 600 -n 400 -q 20 -y 4G -j 6. Demultiplexing of plate barcodes (TruSeq indexes) was automatically done by Illumina software when generating raw FASTQ files. Origin of “Undetermined” reads was analysed using BLAST^50^. Well barcode demultiplexing and separation and extraction of VH and VL sequences was done by looping through merged FASTQ files and list of indexes with custom UNIX script using regular expression. At this point, files were converted into FASTA format. Index matching was done with UNIX tool agrep^51^ to allow one mismatch and variable domain extraction with egrep, both available freely from Ubuntu repositories. Unique sequences from each sample were collapsed and counted with fastx_collapser from FASTX-toolkit^52^. Total sequence count and two most abundant sequence counts were extracted for each sample and used in filtering out samples without Fab sequence (Formula 1) or high sequence background (Formula 2). The UNIX codes and scripts describing the data analysis until filtering can be found at the end of the supplementary materials. Samples where both VH and VL sequences passed the filters were used for candidate selections. Limits for the filters were set based on data from sanger sequencing verification of plate RS016.

1 $$\begin{aligned} \text {Filter}\,1: \frac{Top\,sequence\,count_{sample}}{Total\,sequence\,count_{sample}} > \frac{Top\,sequence\,count_{empty\_control}}{Total\,sequence\,count_{empty\_control}}, \end{aligned}$$

2 $$\begin{aligned} \text {Filter}\,2: \frac{Top\,sequence\,count_{sample}}{Second\,sequence\,count_{sample}} > 2. \end{aligned}$$

Tabulated immunoassay and sequencing data were analysed using Python 3.8 with packages pandas (v1.4.3), numpy (v.1.22.3), scipy (v1.7.3) and visualized using using packages matplotlib (v3.5.1) and seaborn (v0.11.2). DNA sequences were translated using Seq module from Biopython^53^ (v1.79) package and translated amino acid sequences numbered using ANARCI^54^ (v2020.04.23) through AbNumber (v0.2.7). Four-parameter log-logistic function was fitted to EC$_{50}$ data with scipy (v1.8.0) to estimate apparent affinities. All used python packages are freely available from anaconda repositories. Two-tailed Mann–Whitney U test from scipy package was used to compare poor anti-NS1 genotype TRF signals from screening to parental genotype. Five clones from different sublibraries were selected for comparison.

T-test simulation was also done with Python. To generate the data, ttest_ind function from stats module of scipy package was used in loop with randomly generated data with normal distribution using normal function from random module of numpy. Both groups were generated with same standard deviation (calculated from cv) with mean values of 3 and 6 (= twofold difference). Briefly, group sizes from two to 20 were tested with cv ranging from 5 to 50% with 5% intervals. For each sample size : cv combination, data was regenerated 1000 times followed with execution of two-sided t-test and p-values from each iteration collected. Minimum sample size required for statistically significant twofold difference for given variance was calculated from the mean p-values, by setting significance level to p $\le$ 0.05.

### Clone selection, library quality assessment and EC$_{50}$ immunoassays

Clone selections was done based signal to background (S/B) ratio of screening immunoassay by dividing each clones time-resolved fluorescence signal with mean signal from empty controls of the same screening plate. Both blind and genotype-assisted selections of candidate clones from each screened library were done and compared. Genotype for each clone was defined as unique combination of VH and VL sequences, except for anti-NS1 library, where each clone only had one mutation in specified position of CDRH2 or CDRH3. In blind selection, clones excluding the known control wells, were ranked based on the S/B and top 10 or 20 clones were selected and number of unique genotypes counted. In genotype-assisted selection, functional immunnoassay data was grouped by genotype and selections done based on maximum S/B from each genotype group. For retrospective library quality assessment, number of unique genotypes and clone count within the groups were calculated. For anti-NS1 libraries, number of unique amino acid residues in each mutated position was calculated. For comparison, also poorly performing genotypes from anti-NS1 library were selected. Genotypes with more than three clones and with statistically significant difference in screening assay S/B (p < 0.05, two-tailed Mann-Whitney U test) compared to parental were considered as candidates for EC$_{50}$ assay.

EC$_{50}$ TRF immunoassays were concluded on GAH plates (see Supplementary Materials). All incubations were done at room temperature with slow shaking. Briefly, clarified lysates from 5 mL expression cultures (upscaled from 96-well plate expression) were diluted 1:5 (anti-NS1) or 1:10 (anti-DARPin) to AB and 100 $\upmu$L was added to each well. Plates were incubate for 1 h, followed with addition of 100 $\upmu$L biotinylated antigen as triplicates, with exception to two strongest concentrations for which only one well was used. Dilutions of 1000, 500, 50, 25, 5, 1, 0.1, 0.01 and 0.001 ng/mL of bio-DENV2-NS1 and 10,000, 2000, 1000, 500, 250, 75, 10, 1 and 0.1 ng/mL in AB were used for anti-NS1 and anti-DARPin assays, respectively. For poor anti-NS1 binders, two of the lowest concentrations were dropped and two strongest applied as triplicates. After antigen addition, plates were incubated for 2 h, followed by two washes and addition of 100 $\upmu$L of filtered, 100 ng/mL SA-Eu. After 15 min incubation wells were washed two times and 200 $\upmu$L of Delfia enhancement solution was added. Finally, after 10 min incubation the TRF signal was measured using Victor 1420 Multilabel Counter.

### Supplementary Information

Supplementary Information.

## Data Availability

The NGS raw data generated and analyzed during the current study are available in the Sequence Read Archive (SRA), under accession number PRJNA951910. Data related to anti-Dengue NS1 binders are available upon request from the corresponding author.
