# Supplementary material for: Genotyped functional screening of soluble Fab clones enables in-depth analysis of mutation effects
Source: Sci Rep. 2023 Aug 11;13:13107. doi: 10.1038/s41598-023-40241-2 (PMC10421887; doi:10.1038/s41598-023-40241-2)
Supplement: Supplementary file 1 — Supplementary Information. [file 41598_2023_40241_MOESM1_ESM.pdf]

Genotyped functional screening of soluble Fab clones enables in-depth analysis of mutation effects

Sami Oksanen[1,*], Roope Saarinen[1], Anttoni Korkiakoski[1], Urpo Lamminmäki[1], and Tuomas Huovinen[1,]**

[1]Department of Life Sciences, University of Turku, 20520, Turku, Finland
[*]sami.j.oksanen@utu.fi
[**]tuomas.huovinen@utu.fi

ABSTRACT

Monoclonal antibodies (mAbs) and their fragments are widely used in therapeutics, diagnostics and basic research. Although display methods such as phage display offer high-throughput, affinities of individual antibodies need to be accurately measured in soluble format. We have developed a screening platform capable of providing genotyped functional data from a total of 9 216 soluble, individual antigen binding fragment (Fab) clones by employing next-generation sequencing (NGS) with hierarchical indexing. Full-length, paired variable domain sequences (VL-VH) are linked to functional screening data, enabling in-depth analysis of mutation effects. The platform was applied to four phage display-selected scFv/Fab screening projects and one site-saturation VH affinity maturation project. Genotyped functional screening simultaneously enabled the identification of affinity improving mutations in the VH domain of Fab 49A3 recognizing Dengue virus non-structural protein 1 (NS1) serotype 2 and informed on VH residue positions which cannot be changed from wild-type without decreasing the affinity. Genotype-based identification revealed to us the extent of intraclonal signal variance inherent to single point screening data, a phenomenon often overlooked in the field. Moreover, genotyped screening eliminated the redundant selection of identical genotypes for further study and provided a new analysis tool to evaluate the success of phage display selections and remaining clonal diversity in the screened repertoires.

Supplementary Material

Preparation of goat anti-human (Fab specific) (GAH) plates

GAH plates were prepared by passive coating on C12 MaxiSorp plates (Thermo Fisher Scientific, USA) by first diluting the IgG 1:1 to 0.9 % saline solution, then added to solution containing 30 mM HCl and incubating the mixture at room temperature for 5 minutes. The acid-IgG solution was then mixed with 0.1M NaH_2PO_4, followed by pipeting 200 ng of IgG per well. Coating reaction was incubated at 35°C o/n in sealed box with moisturized paper towels. Wells were washed twice with Delfia wash solution (Kaivogen Oy, Finland) and then filled with saturation buffer (50 mM NaH_2PO_4, 0.9 % NaCl, 6 % sorbitol, 85 µM $CaCl_2$, 0.05 % Germal II, 0.1 % filtered BSA and 4 µM EDTA. Saturation reaction was incubated at RT o/n in sealed box with moisturized paper towels. Next morning the wells were aspired empty and allowed to air dry before storage at +4C, closed bags with dessicants.

Preparation of anti-SpyCatcher and anti-SdyCatcher Fab libraries for screening

Selection of anti-SpyCather scFv clones by phage display

The anti-SpyCatcher and anti-SdyCatcher Fab molecules were generated in the Department of Life Technologies at the University of Turku by phage display. Synthetic antibody phage display libraries ScFvM and ScFvP[1] were selected according to standard protocol for three rounds against SpyCatcher protein.

On the first round of panning, 5 x 10^{12} cfu of scFv-phage particles (1:1 molar mix of scFvM and scFvP libraries) were incubated for 1 h at RT on rotation with 500 µg of paramagnetic microbeads precoated to saturation with SpyCatcher protein (Dynabead M-270Epoxy, Invitrogen, USA; coating performed according to manufacturer´s instructions) in 2mL assay buffer RED (Kaivogen Oy, Finland; 50 mmol/L Tris-HCl pH 7.75; 150 mmol/LNaCl; 0.05 % NaN3; 0.01 % Tween-40; 0.05 % bovine gamma-globulin; 20 µm/L diethylenetriamine penta-asetate; 0.5 % bovine serum albumin; 20 mg/L cherry red). The second round of panning was performed similarly to the first round, except that 1 x 10^{11} cfu of scFv-phages from the 1st round were used as the library input. All binding reactions were washed three times with 1 mL Kaivogen wash buffer using DynaMag-2 bar magnet(Invitrogen, USA) to collect the beads. Trypsin (Sigma Aldrich, USA) was used for the phage elution and XL-1 Blue cells (Stratagene, USA) for phage rescue as described in[1]. On panning rounds one and two, a negative selection step was included by preincubating the scFv-phage libraries with BSA-coated microbeads (equivalent amount of beads as for positive

selection) for 1 h, collecting the beads with DynaMag-2 bar magnet and transferring the supernatant to the positive panning reaction.

The third round of selection was performed by providing the antigen, anti-digoxigeninscFv-SpyCatcher clone 203A2 (Department of Life Technologies, University of Turku) in solution at 2.5 nM concentration and mixed with 1 x 10^{10} cfu 2nd round phages in 2 mL assay buffer RED. The binding reaction was incubated for 30 min at RT on rotation, after which, 50 µg of paramagnetic M280 streptavidin Dynabeads (Invitrogen, USA) precoatedto saturation with biotin-S-S-digoxigenin conjugate (Department of Life Technologies, University of Turku) were added to the reaction. The incubation was continued for 30 min on rotation followed by washes, elution and phage rescue as above. The following day, a DNA miniprep was prepared from the cell mass harbouring the 3rd round output pEB32x-scFv phagemids.

Conversion of scFv output library into Fab input library by selective RCA

The enriched anti-SpyCatcher scFvs from the 3rd round were converted into Fab format with selective RCA[2]. A VL-CL-VH -mega primer was prepared by amplifying VL and VH domain genes from the 3rd round pEB32x-scFv anti-SpyCatcher miniprep DNA with primer pairs HS012/WO375 and HS015/HS034 using Phusion II HSDNA polymerase (Thermo, USA) according to manufacturer´s instructions, respectively. Similarly, CL domain was amplified using codon-harmonized Fab construct pEB32x hrFab0 miniprep DNA[3] as the template with primers EB197 andHS014. All PCR reactions were amplified with the thermal cycling protocol: (1) init.den. at 98 °C for 30 s, (2) den. at 98 °C for 5 s, (3) ann. at. 60 °C for 15 s, (4) ext. at 72°C for 25 s (a total of 25 cycles, steps 2-4) and (5) final ext. at 72 °C for 5 min. The PCR products were purified with GeneJet PCR purification kit (Thermo, USA) and 500 ng of purified products were digested with 10 U of LguI in 20 µl reaction in 1x Tango bufferovernight at 37 °C.

The LguI reactions were purified as above and a triple fragment ligation was set up containing 50 ng of each of the LguI digested fragments, 1 x T4 DNA ligase buffer and5 U of T4 DNA ligase (Thermo, USA) in a total volume of 20 µl. The ligation was incubated overnight at 16 °C followed by two consecutive PCR reactions to amplify the VL-CL-VH fragments. The first PCR reaction contained 5 µl sample of the ligation as template in a total reaction volume of 200 µl using primers WO375 and HS034. The PCR reaction was purified as above and a 2.5 µl sample was taken as the template for the second asymmetric PCR reaction performed in a 100 µl volume. The latter reaction contained only primer WO375. Both reactions were thermally cycled as above with Phusion II HSDNA polymerase (except for 30 cycles instead of 25). The PCR reactions were purified with GeneJet PCR purification kit and 750 ng of the ssDNA PCR product (contains a mix of ss and dsDNA) was 5´-phosphorylated in a 30 µl reaction volume containing 1 x T4 PNK buffer and 15 U of T4 polynucleotide kinase (Thermo, USA). The reaction was incubated overnight at 37 °C and stored frozen at -20 °C for further use. Selective RCA was performed as described earlier[2]. The final product, pEB32x-Fab anti-SpyCatcher library, was transformed to SS320 cells by electroporation. Illustration of the panning scheme can be seen in Figure S15.

Selection of anti-SpyCatcher Fab clones by phage display

A Fab display phage stock was prepared from the SS320 cells harbouring pEB32x-Fab anti-SpyCatcher phagemid DNA with VCS-M13 (Stratagene, USA) superinfection according to standard protocol. The Fab-phages were selected against in vivo biotinylated SpyCatcher (produced in BL21/ BirA+ E. coli strain and purified in Department of Life Technologies, University of Turku) and SdyCatcher.

First round of selection against in vivo biotinylated SpyCatcher and SdyCatcher proteins were performed in parallel. First, a negative selection step was done by preincubating 5 x 10^{10} cfu phages from the generated stock with 100 µg of M280 streptavidin Dynabeads for 1 h, collecting the beads with DynaMag-2 bar magnet and transferring half of the supernatant to the positive panning reaction (other half used as control reaction with no antigen on beads). The positive selections were done by incubating collected phages with 100 µg of M280 strepavidin Dynabeads saturated with the biotinylated Catchers in1 mL of TBT-0.05 (50 mM Tris, 150 mM NaCl, 1 % BSA, 0.05 % Tween-20, pH 7.5).The binding reactions were incubated for 1 h at RT on rotation, washed two times on bar magnet with TBT-0.05 and once with TBS (50 mM Tris, 150 mM NaCl, pH 7.5), eluted with trypsin and rescued as above. DNA minipreps were extracted from scraped XL-1 Blue cell mass (GeneJet Plasmid miniprep kit, Thermo, USA) harbouring enriched pEB32x-Fab anti-SpyCatcher and anti-SdyCatcher repertoires.

On second selection round, 30 µg of neutravidin coated Dynabeads (Department of Life Technologies, University of Turku) saturated with in vivo biotinylated Catchers were incubated with 1 x 1010 phages from 1st round Fab-phage stocks in 1 mL of TBT-0.05. The binding reactions were incubated for 1 h at RT on rotation, washed twice on bar magnet with TBT-0.1 (50 mM Tris, 150 mM NaCl, 0.1 % Tween-20, pH 7.5) and once with TBS, eluted with trypsin and rescued as above. DNA minipreps were extracted from scraped XL-1 Blue cell mass (GeneJet Plasmid miniprep kit, Thermo, USA).

Third round of selections against in vivo biotinylated SpyCatcher and SdyCatcher were performed in parallel by saturating M280 streptavidin Dynabeads with biotinylated Catchers (50 µg beads / binding reaction) and providing 5 x 10^{10} cfu phages from the 2nd round Fab-phage stocks in 1 ml of assay buffer RED. The binding reactions were incubated for 30 min at RT on rotation, washed on bar magnet, eluted with trypsin and rescued as above. DNA minipreps were extracted from scraped

XL-1 Blue cell mass(GeneJet Plasmid miniprep kit, Thermo, USA) harbouring enriched pEB32x-Fab anti-SpyCatcher and anti-SdyCatcher repertoires and cloned with SfiI to pAK400 vector for screening. 10 + 10 plates were screened from both Fab libraries.

Preparation of anti-NS1 affinity maturation library for screening

For affinity maturation of anti-NS1 Fab 49A3, total of 20 sublibraries were generated using site-saturation mutagenesis PCR (Figure S16 a). For this purpose, total 20 forward NNK-primers, each targeting single codon either in CDRH2 or CDRH3, were designed and ordered from Sigma Aldrich, USA. Mutated Fab fragments ("A") were generated with PCR with NNK-primers and pAKrev2. Non-mutated Fab fragments ("B"), containing the rest of the template Fab sequence and ovelapping sequence with corresponding fragment A, were amplified using WO375 and one of fragment B primers (Sigma), designed earlier for another project. Both fragment A and B PCR reactions consisted of 10 ng of template plasmid DNA, 0.02 U/µL Phusion High-Fidelity DNA Polymerase (Thermo Fisher Scientific, USA), 1 x HF Buffer, 200 µM dNTP, and 0.5 µM primers and thermal cycling was 30 cycles of 98°C (30s), 57°C (30s) and 72°C (35s) with a 98°C (30s) initial denaturing at the beginning and 72°C (5min) final elongation step at the end. The amplification was verified with gel electrophoresis on 1 % agarose gel and purified from cut gel slabs using GeneJet Gel extraction kit (Thermo Fisher Scientific). Fab cassettes of sublibraries were then assembled via PCR by mixing A and B fragments in 1:1 molar ratio and amplifying them with WO375 and pAKrev2 primers. The PCR protocol for assembly was the same as before, except of increasing annealing temperature to 58°C. The used primer sequences and fragment pairs are shown in Table S1. The used site-saturation mutagenesis PCR is illustrated in Figure S16 b. Casettes were then cloned into pAK400 expression vector for screening by SfiI (Thermo) digestion of casette and vector, followed by ligation with T4 DNA ligase (Thermo), according to manufacturers protocol.

Preparation of anti-DARPin Fab library for screening

Anti-DARPin Fab generation was started by enriching DARPin binding antibodies in scFv format from mixed scFv M and P libraries according to standard protocol for three selection rounds[1]. The targets for the selection were biotinylated anti-lysozyme DARPin clones 378 (1st and 2nd rnd) and 398 (3rd rnd), both containing three 33 aa diversity repeats between N- and C-cap repeat. First two rounds were performed by coating DARPins to saturation on paramagnetic M280 streptavidin beads (Invitrogen) and the third round was performed by providing 1 nM and 100 pM biotinylated DARPin in solution and capturing the phage-DARPin complexes on paramagnetic streptavidin beads.

Phagemid DNA pEB32x-scFv was extracted from output cells with a miniprep kit (GeneJET, Thermo) and used as template to amplify VL and VH domains by PCR. The resulting amplicons were digested with LguI and joined to CL domain containing compatible cohesive ends with T4 DNA ligase (Thermo). The VL-CL-(RBS-PelB-)VH fragment was PCR amplified from the ligation and joined to preamplified and gel extracted (GeneJET, Thermo) CH1 domain fragment by assembly PCR. The resulting Fab fragment was amplified by PCR and cloned with SfiI (Thermo) sites to pEB32x vector for Fab selections. Phusion DNA polymerase was used in all PCR reactions according to manufacturers instructions and list of oligos used for the construction are listed in Table S2.

Fab Phage stock was prepared by transforming DNA pEB32x-Fab to SS320 cells by electroporation and preparing phage display stock from 20 mL culture according to standard protocol. Fab phage display selections were carried out as above for scFv selections for rounds one and two. Third round was performed by incubating Fab phage in microtiter wells coated with DARPin anti-GFP 3G86.32[4] fused to SNAP-tag. Final 4th round of selection was performed by providing in solution 10 nM and 1 nM biotinylated DARPin-SpyCatcher, in which the N-terminal DARPin was a polyclonal sample from a binary library containing Tyr or Ser at selected paratope positions[5]. The antigen-phage complexes were captured from buffer on paramagnetic streptavidin beads for washing and elution. The fourth round output was cloned to pAK400 vector with SfiI sites for indexing and single Fab clone screening.

Shuffling of Fab sequences to create more DNA diversity was performed between rounds 1 and 2 by performing staggered extension PCR process (StEP)[6]. StEP was performed using FirePol DNA polymerase (Solis BioDyne), 3 ng/µl of phagemid DNA in 100 µl reactions with primers WO375 and TH366. Different extension times and temperatures were used in parallel for shuffling. Fab fragments amplified in all four PCR reactions performed in different conditions were pooled after gel extraction, SfiI digested and cloned back to pEB32x with SfiI sites for 2nd round of Fab phage display selection. Illustration of the panning scheme can be seen in Figure S17.

Preparation of anti-SARS CoV-2 Nucleocapsid Protein (anti-NP) Fab libraries for screening

Selection of anti-NP scFv clones by phage display

The anti-NP Fab molecules were generated in the Department of Life Technologies at the University of Turku by phage display. Synthetic antibody phage display libraries ScFvM and ScFvP[1] were selected according to standard protocol for four rounds against biotinylated SARS CoV-2 Nucleocapsid Protein with GST-tag. Antigen was kindly provided by P. Jalkanen and P.Kolehmainen from prof. Julkunen's group (Department of Virology, University of Turku), expressed in Sf9 insect cells and

Table S 1. Primers used in anti-NS1 affinity maturation

Primer name	Sequence (5'->3')	Fragment pair
Fragment A primers (NNK)		
TH302_NS1H2F1	aagggtctcgagtgggtcagtNNKatcgctccttctggtgggag	A
TH303_NS1H2F2	aagggtctcgagtgggtcagtttgatcNNKccttctggtgggagcacg	A
TH304_NS1H2F3	aagggtctcgagtgggtcagtttgatcgctNNKtctggtgggagcacgaattatg	A
TH305_NS1H2F4	aagggtctcgagtgggtcagtttgatcgctcctNNKggtgggagcacgaattatg	A
TH306_NS1H2F5	cagtttgatcgctccttctNNKgggagcacgaattatgcagatag	B
TH307_NS1H2F6	cagtttgatcgctccttctggtgggNNKacgaattatgcagatagcgtgaa	B
TH308_NS1H2F7	cagtttgatcgctccttctggtgggagcNNKaattatgcagatagcgtgaagggtc	B
TH309_NS1H2F8	cagtttgatcgctccttctggtgggagcacgNNKtatgcagatagcgtgaagggtc	B
TH313_NS1H3F1	gacactgccgtctactactgtNNKcgtgaccatcagtacgcttacg	C
TH314_NS1H3F2	gacactgccgtctactactgtgctNNKgaccatcagtacgcttacggtg	C
TH315_NS1H3F3	gacactgccgtctactactgtgctcgtNNKcatcagtacgcttacggtgactac	C
TH316_NS1H3F4	gacactgccgtctactactgtgctcgtgacNNKcagtacgcttacggtgactactgg	C
TH317_NS1H3F5	gacactgccgtctactactgtgctcgtgaccatNNKtacgcttacggtgactactgg	C
TH318_NS1H3F6	ctactgtgctcgtgaccatcagNNKgcttacggtgactactggggtc	D
TH319_NS1H3F7	ctactgtgctcgtgaccatcagtacNNKtacggtgactactggggtc	D
TH320_NS1H3F8	ctactgtgctcgtgaccatcagtacgctNNKggtgactactggggtcaggg	D
TH321_NS1H3F9	tcgtgaccatcagtacgcttacNNKgactactggggtcagggtacac	E
TH322_NS1H3F10	tcgtgaccatcagtacgcttacggtNNKtactggggtcagggtacactagtc	E
TH323_NS1H3F11	tcgtgaccatcagtacgcttacggtgacNNKtggggtcagggtacactagtc	E
TH311_NS1_H62_F	gtgggagcacgaattatgcagatNNKgtgaagggtcgcttcaccatc	F
Fragment B primers		
TH276_SKA_H2R1	actgacccactcgagaccctt	A
TH310_NS1_H2R2	agaaggagcgatcaaactg	B
TH290_SKA_H3R1	acagtagtagacggcagtgtc	C
TH324_NS1_H3R2	ctgatggtcacgagcacagtag	D
TH325_NS1_H3R3	gtaagcgtactgatggtcacga	E
TH312_NS1_H62_R	atctgcataattcgtgctcccac	F
End primers		
WO375	TCACACAGGAAACAGCTATGAC	
pAKrev2	CCGTGACGCAGTAGCGGTAAACG	

Table S 2. Oligos used in Fab assembly

Primer	Template DNA	Sequence
WO375	VL/Fab	TCACACAGGAAACAGCTATGAC
HS012	VL	ACCATTGCTAGCTCTTCTCGACTTTCGTCCCCTGGC
HS015	VH	ACCATTGCTAGCTCTTCAGCTTGAAAGCGGCGGTGG
HS034	VH/Fab	GCCGAGCTCACGGTGACTA
EB197	CL	ACCATTGCTAGCTCTTCGTCGAGATTAAACGGACTGTGGC
HS014	CL	ACCATTGCTAGCTCTTCAAGCAGCTGTACCTCAGCC
TH252	CH1	GCGTCTACCAAAGGCCCTTC
HS031	CH1	GATGGTGATGATGGTGATCGG
TH366	Fab-p3Δ	ACCAGAACCGCCACGACCTTCAA

purified using the GST tag. Biotinylation was done with EZ-Link NHS-PEG_4-Biotin with 12 x molar excess of biotin, according to the manufacturers protocol. Assay buffer RED (Kaivogen Oy, Finland; 50 mmol/L Tris-HCl pH 7.75; 150 mmol/LNaCl; 0.05 % NaN3; 0.01 % Tween-40; 0.05 % bovine gamma-globulin; 20 µm/L diethylenetriamine penta-asetate; 0.5 % bovine serum albumin; 20 mg/L cherry red, later referred as AB) was used as panning buffer. Three separate phage display selections were conducted in parallel, each with different additives in AB ; triton-BSA ("N1"), triton-saliva ("N2") or no additives ("N3"). All binding reactions were washed with TBT-0.5 (50 mM Tris, 150 mM NaCl, pH 7.5, 0.5% Tween-20) using DynaMag-2 bar magnet (Invitrogen, USA) to collect the beads. Trypsin (Sigma Aldrich, USA) was used for the phage elution and XL-1 Blue cells (Stratagene, USA) for phage rescue as described earlier[1].

On the first round of panning, 5 x 10^{12} cfu of scFv-phage particles (1:1 molar mix of scFvM and scFvP libraries) were incubated for 1 h at RT on rotation with 500 µg of paramagnetic M280 streptavidin Dynabeads precoated to saturation with bio-N-GST (Invitrogen, USA; coating performed according to manufacturer´s instructions) in 2mL AB.

The second round of panning was performed similarly to the first round, except that 1 x 10^{11} cfu of scFv-phages from the 1st round were used as the library input and smaller amount of streptavidin beads were used (50 µg). Also, a negative selection step was included by preincubating the scFv-phage libraries with non-coated streptavidin beads (equivalent amount of beads as for positive selection) for 1 h, collecting the beads with DynaMag-2 bar magnet and transferring the supernatant to the positive panning reaction.

The third and fourth round of selection were performed by providing the antigen in solution at 1 nM concentration and mixed with 1 x 10^{10} cfu 2nd or 3rd round phages in 2 mL AB. The binding reaction was incubated for 30 min at RT on rotation, after which, 50 µg of paramagnetic M270 Epoxy beads (Invitrogen, USA), coated with Avidin (Department of Life Technologies, University of Turku) were added to the reaction. The incubation was continued for 10 min on rotation followed by five washes, elution and phage rescue as above. After fourth panning round, DNA minipreps were prepared from the cell mass harbouring the 4th round output pEB32x-scFv phagemids.

Fab conversion of panned anti-NP scFv libraries

The enriched anti-NP scFvs from the 4th round were converted into Fab format with a modified version of FASTR-method[7]. All of the used primer sequences can be found from Tables S2 and S1. Briefly, VL, CL, VH and CH1 domains of antibody were amplified with primers with LguI recognition site in overhang. VL and VH domains were amplified from the 4th round pEB32x-scFv anti-NP minipreps with primer pairs WO375/HS012 and HS015/HS016 using FIREPol DNA polymerase (Solis Biodyne, Estonia) according to manufacturer´s instructions, respectively. Similarly, CL and CH1 domains were amplified using Fab construct 001OHB12 pAK400-Fab miniprep, originating from hrFab0[3] as the template with primers pairs EB197/HS014 and EB197/pAKrev2, respectively. All PCR reactions consisted of 50 ng of template plasmid DNA, 0.05 U/µL FIREPol DNA polymerase, 1 x BD Buffer, 250 µM dNTP, and 0.1 µM primers and thermal cycling was 35 cycles of 95°C (30s), 60°C (30s) and 72°C (30s) with a 95°C (5min) initial denaturing at the beginning and 72°C (4min) final elongation step at the end. The PCR products were purified with Zymo DNA Clean & Concentrator-5 kit (Zymo Research, USA) and 400 ng of purified products were digested with 10 U of LguI (Thermo, USA) in 20 µl reaction in 1x Tango bufferovernight at 36°C.

The LguI reactions were purified as above and a four fragment ligation was set up containing 20 ng of each of the LguI digested fragments, 1 x T4 DNA ligase buffer and 5 U of T4 DNA ligase (Thermo, USA) in a total volume of 20 µl. The ligation was incubated at room temperature for one hour. The ligation products were used directly for PCR with end primers WO375/pAKrev2 to amplify the complete Fab casette. Correct amplificatin was verified by running a small amount of PCR products on 1 % agarose gel at 90 V for 40 minutes. PCR products were again purified with Zymo kit as above, followed with digestion with SfiI (Thermo) in 20 µL reaction consisting of 1 x buffer G, 20 U of SfiI and 2 µg of Fab library DNA. Digestion was incubated for 2 hours at 50°C, followed by gel extraction, after running the digestion products on 1 % agarose gel at 80 V for 40 minutes, using GeneJet Gel extraction kit (Thermo) to remove any background. The digested products were cloned into pEB32x vector to using T4 DNA ligase, same as above. The libraries in display vectors were transformed to XL1 cells by electroporation.

Selection of anti-NP Fab clones by phage display

A Fab display phage stocks for the three separate libraries were prepared from the XL1 cells harbouring pEB32x-Fab anti-NP phagemid DNA with VCS-M13 (Stratagene, USA) superinfection according to standard protocol. The Fab-phages were again selected against in biotinylated SARS-CoV-2-N. No additives were added to AB for any of the three libraries in Fab phage selections.

In first round of selection against in biotinylated N-GST a negative selection step was included by preincubating 20 µL of phages from the generated stock with mixture of M280 streptavidin Dynabeads, M270 Epoxy Dynabeads and M270 Neutravidin Dynabeads (beads from Invitrogen, USA, coated at Department of Life Technologies, University of Turku), 100 µg of each, for 1 h, collecting the beads with DynaMag-2 bar magnet and transferring half of the supernatant to the positive panning reaction (other half used as control reaction with no antigen on beads). The positive selections were done by incubating collected phages

with 100 µg of M280 strepavidin Dynabeads saturated with the biotinylated antigen in 1 mL of AB. The binding reactions were incubated for 30 min at RT on rotation, washed two times on bar magnet while transferring beads to new tube between washes, eluted with trypsin and rescued as above. DNA minipreps were extracted from scraped XL-1 Blue cell mass (GeneJet Plasmid miniprep kit, Thermo, USA) harbouring enriched anti-NP repertoires.

On second selection round, more stringent panning was done by shorter incubation times, increased number of washes and lower concentration of antigen in solution. In addition, blocking with free GST was included to decrease the amount of GST-specific binder enrichment. 10 µL of phages from 1st round were incubated in 1 mL of AB with 100 pM bio-N-GST and 1 nM free GST and incubated for 15 minutes RT in rotation. Antigen bound phages were captured by adding 100 µg of M280 Streptavidin Dynabeads to the reaction, incubating 10 min RT, rotation and washing the beads with TBT-0.5 five times on magnet, transfering beads to new tubes between washes. Also, control reaction without antigen was included with similar steps. Elution with trypsin, rescue and minipreps were done as above.

For "NP-2" screening project, the Fab libraries from round 1 and 2 were cloned to pAK400 expression vector using SfiI restriction enzyme and T4 DNA ligase and transformed into XL1 cells for screening, as described earlier.

For "NP-1" screening project, a capture Fab for sandwich panning was screened from round 2 libraries cloned into pLK06H (Fab fusion with alkaline phosphatase) expression vector in XL1 cells, plated on agar plates supplemented with 1 % D-glucose and 100 µg of ampicillin. Briefly, 90 clones from each of the three 2nd round libraries were inoculated to 200 µL of SB (30 g/L Tryptone, 20 g/L Yeast extract, 10 g/L MOPS) supplemented with 1 % D-glucose, 100 µg of ampicillin and 10 µg of tetracyclin on 96-well plate with 3 x wells with control anti-N Fab CR3009[8] (gene purchased from Twist Bioscience, USA, produced in XL1 similarily as described in this article) and 3 x empty control wells and incubated at 37°C, 900 rpm o/n. Next morning, cells from primary culture were inoculated with four dips with 96-well replicator into fresh 200 µL of SB with 0.05 % D-glucose and antibiotics, incubated for four hours at 37°C, 900 rpm. Protein production was induced by addition of IPTG to final concentration of 200 µM, after which temperature was expression was allowed at 26°C, 900 rpm for 16 hours. Cells from original culture plates were stored at -70°C in 16 % glycerol. Cells from expression were lysed by addition of 20 µL of 10 x lysis buffer (10 mg/mL lysozyme from chicken egg white L6876 (Sigma Aldrich, United Kingdom), 25 U/µL Pierce Universal Nuclease (Thermo Fisher Scientific, USA) in PBS (20 mM sodium phosphate, 300 mM sodium chloride, pH 7.4), followed by 30 min incubation in room temperature in slow agitation, and finally freezing at -70°C.

All dilutions for immunassays were done using Assay Buffer Red and washes using Kaivogen Wash buffer, both purchased from Kaivogen. Delfia Plate Wash (Wallac, Turku Finland) instrument was used for plate washing. All incubations were done at room temperature with slow shaking. Lysate was clarified by centrifugation before the immunoassay. Clear streptavidin plates (Kaivogen Oy, Finland) were prewashed, followed by addition of 100 µL bio-N-GST with concentrations of 0, 0.3, 3, 6, 12 and 120 nM in AB and incubation of 30 minutes. Wells were washed 4 times and lysate diluted 1:5 in AB was added to each well. After 1 hour of incubation, wells were again washed 4 times, followed by addition of 100 µL pNPP buffer (1mg/mL pNPP, 500 mM Tris, 200 mM NaCl, 10 mM $MgCl_2$, pH 9.0). Absorbance at 405 nm was measured after 1 hour incubation using Victor 1420 Multilabel Counter (Wallac, Finland). As there was no real signal saturation for any of the clones, 12 clones with highest specific absorbance were selected for EC_{50} immunoassays. For the assay, expression of these clones was done similarly as before, but instead of 200 µL volume, on 5 mL with shaking speed of 300 rpm, inoculated from glycerol preps made earlier. Fab concentration was measured from clarified lysates diluted 1:5 to pNPP buffer with NiNTA and size exclusion chromatography purified CR3009 Fab-AP standard series.

To compare the apparent affinities against of selected clones, immunoassay on clear streptavidin plates were again carried out. 1 nM bio-N-GST was added on prewashed wells and incubated for 30 minutes, followed with 4 washes. 10 mM Fab-AP from lysates were added on wells, in addition to two controls for each clone with either 100 nM free GST (to see whether the Fab binding is dependent on the tag) or well without antigen to measure background. After 30 min incubation the wells were again washed 4 times, followed by addition of pNPP buffer and incubation of 30 min, after which absorbance at 405 nM was measured with Victor 1420 Multilabel Counter. Based on the the immunoassay results, clone from round 2 N1 library ("N1G1) was selected as capture. For this purpose, the Fab gene was cloned in pAK400 expression vector, transformed back into XL1 and expressed in 1 L culture with similar protocol as described above. Purification of the his-tagged Fab was done first with HisPur NiNTA Resin according to manufacturers protocols, and then further purified with size exclusion chromatography instrument Äkta Explorer using HiLoad 26/60 FastFlow Superdex 200 column (GE Healthcare, USA). To use the Fab as capture in panning and immunoassays, biotinylation was done using EZ-Link NHS-PEG_4-Biotin with 20 x molar excess of biotin, according to the manufacturers protocol.

For "NP-1" screening project, a new panning from round 0 N1, N2 and N3 Fab phage libraries was started (sandwich-panned libraries named S1, S2 and S3, respectively).

First round of panning was done with Streptavidin M280 Dynabeads saturated with bio-N1G1 capture Fab. 10 µL of round 0 phages were added to 1 mL of AB with 1 nM N-GST with presence of 10 nM free GST as blocker and incubated for 20 min, rt in rotation. Control reactions were included, similarly to above. 100 µg of capture beads were added and reaction incubated

for another 20 minutes rt, rotation, followed by 3 x washes with TBT-0.5 and once with TBS (50 mM Tris, 150 mM NaCl, pH7.5) on magnet while changing to new tubes. Elution with trypsin, rescue and minipreps were done as above.

Second round of panning was done on solid phase without presence of blocker GST. 100 µg of Streptavidin M280 Dynabeads were first saturated with bio-N1G1 capture Fab by 30 min incubation rt, rotation, washed 3 times, followed by saturation with N-GST with similar incubation and washing. 10 µL of round 1 phages were added to the reaction, followed similar incubation and washes as on round 1. Elution with trypsin, rescue and minipreps were done as above. Fab genes were cloned into pLK06H expression vector for screening, similarly as done for "NP-2". Due to complexity of the panning process, the panning is illustrated in Figure S18.

Control Fab sequences

The amino acid sequences of the Fabs used as positive controls are are shown below in Table S3. All clones share the same constant domain sequences (CH1 and CL). S001E03 was used in anti-SpyCatcher project and was discovered during initial testing of the screening platform. 248G12 was used in anti-DARPin screening, and was discovered in during earlier, unpublished project at University of Turku. N1G1 was the control Fab for anti-SARS-CoV2 projects, discovered similarly to S001E03, during the initial testing of the screening assays.

Table S 3. Amino acid sequences of the Fabs used as positive controls on screening plates

Name	AA sequence 5'->3'
CH1 with His6-tag	ASTKGPSVFPLAPSSKSTSGGTAALGCLVKDYFPEPVTVSWNSGALTSGVHTFPAVLQ SSGLYSLSSVVTVPSSSLGTQTYICNVNHKPSNTKVDKKVEPKSSAATGADHHHHHH
CL	TVAAPSVFIFPPSDEQLKSGTASVVCLLNNFYPREAKVQWKVDNALQSGNSQESVTEQ DSKDSTYSLSSTLTLSKADYEKHKVYACEVTHQGLSSPVTKSFNRGES
S001E03 VH	EVQLLESGGGLVQPGGSLRLSCAASGFTFSSYAMHWVRQTPGKGLEWVSEISPYNGYT TYADSVKGRFTISRDNSKNTLYLQMNSLRAEDTAVYYCARAIPIISQGGVAYYFDYWG QGTLVTVSS
S001E03 VL	EIVLTQSPGTLSLSPGERATLSCRASQSVSSSYLHWYQQKPGQAPRLLIYGASSRATG VPDRFSGSGSGTDFTLTISRLEPEDFAVFYCQQHYSTPWTFGQGTKVEIKR
248G12 VH	EVQLLESGGGLVQPGGSLRLSCAASGFTFSRYGMHWVRQAPGKGLEWVSYITGYGGYT YYADSVKGRFTISRDNSKNTLYLQMNSLRAEDTAVYYCARGRGKAPMDYWGQGTLVTVSS
248G12 VL	EIVLTQSPGTLSLSPGERATLSCRASQSVSSSYLNWYQQKPGQAPRLLIYGASSRATG VPDRFSGSGSGTDFTLTISRLEPEDFAVYYCQQGTYYPPTFGQGTKVEIKR
N1G1 VH	EVQLLESGGGLVQPGGSLRLSCAASGFTFSDYYMHVRQAPGKGLEWVSEINPSSGNTD YADSVKGRFTISRDNSKNTLYLQMNSLRAEDTAVYYCAREYEEHGYYLTDYYFDYWGQ GTLVTVSS
N1G1 VL	EIVLTQSPGTLSLSPGERATLSCRASQSVSSSALAWYQQKPGQAPRLLIYGASSRATG VPDRFSGSGSGTDFTLTISRLEPEDFAVYYCQQMHSTPWTFGQGTKVEIKR

Figures

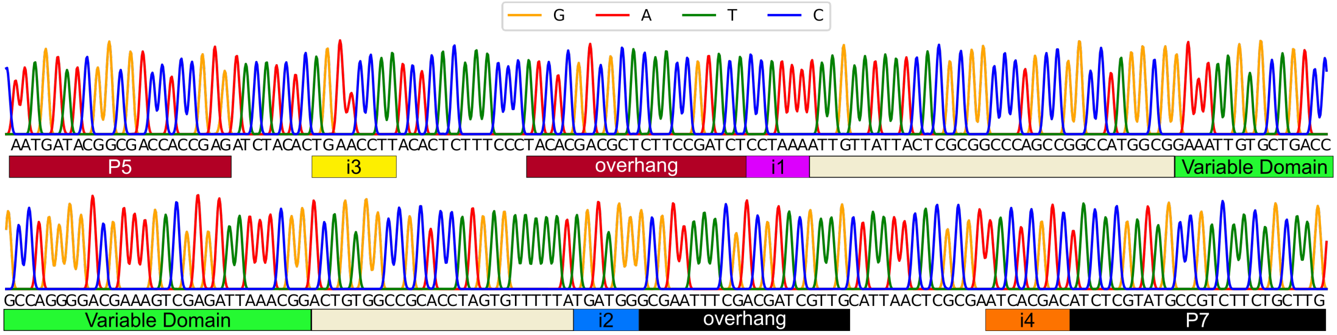

Figure S 1. Annotated Sanger sequencing chromatogram of fully indexed anti-Mc 226A02 VL sequence. From inside, correct custom well indexes i1 and i3 were verified. In addition, Illumina TruSeq i501 (beginning) and i701 (end) adapter sequences were correct and contain the plate indexes i3 and i4 and the flow-cell-hybridizing grafts P5 and P7.

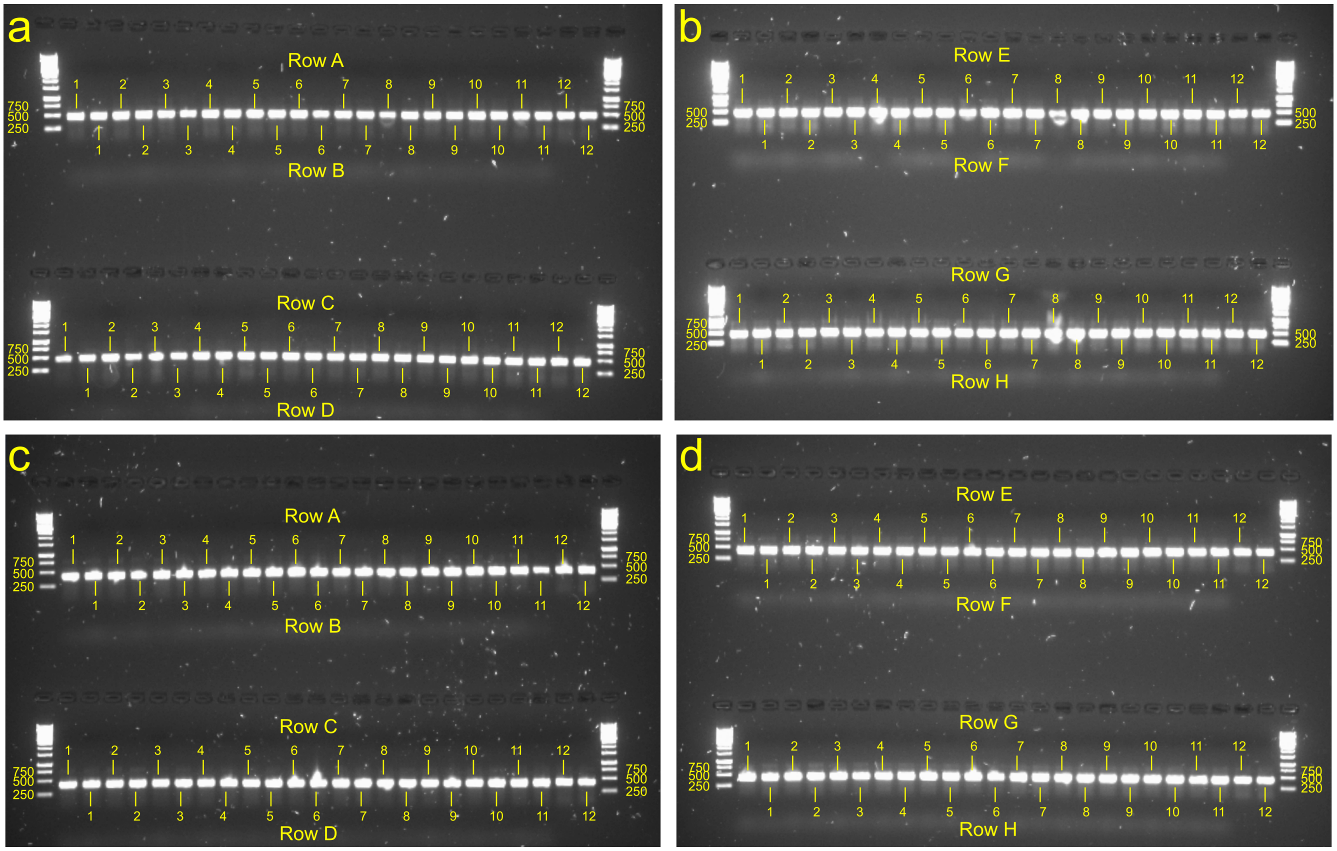

Figure S 2. Annotated electrophoresis gel images of custom well indexing primer testing. (**a-b**) Amplification with VH-indexing primers. (**c-d**) Amplification with VL-indexing primers. Visible bands can be observed on each lane, indicating amplification of variable domains with all 96 primer pairs. 1 kb GeneRuler ladder (Thermo Scientific) was used to confirm the correct size of amplicons (∼500 bp).

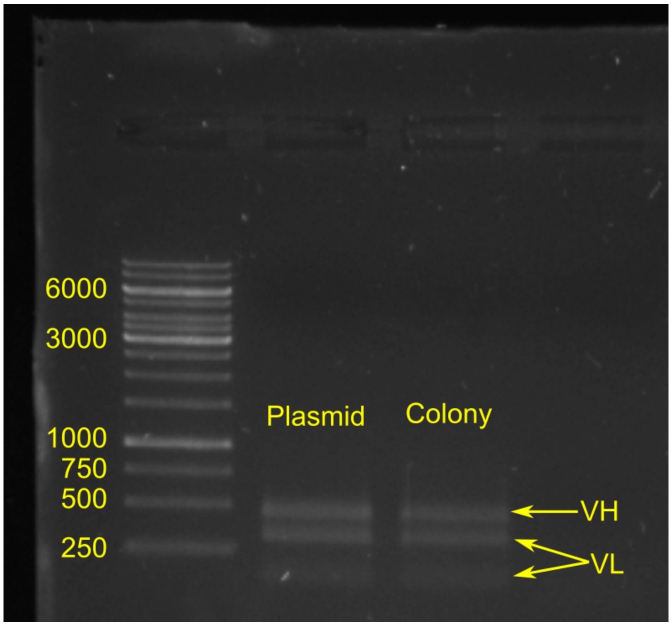

Figure S 3. Annotated electrophoresis gel image of well barcoding PCR of both VL and VH in same reaction, using 10 µL reaction volume. Correct sized bands can be seen for both VH (496 bp) and PstI digested VL (308 + 131 bp) with both plasmid and bacterial colony as template. 1 kb GeneRuler ladder (Thermo Scientific) was used to confirm the correct size of the products.

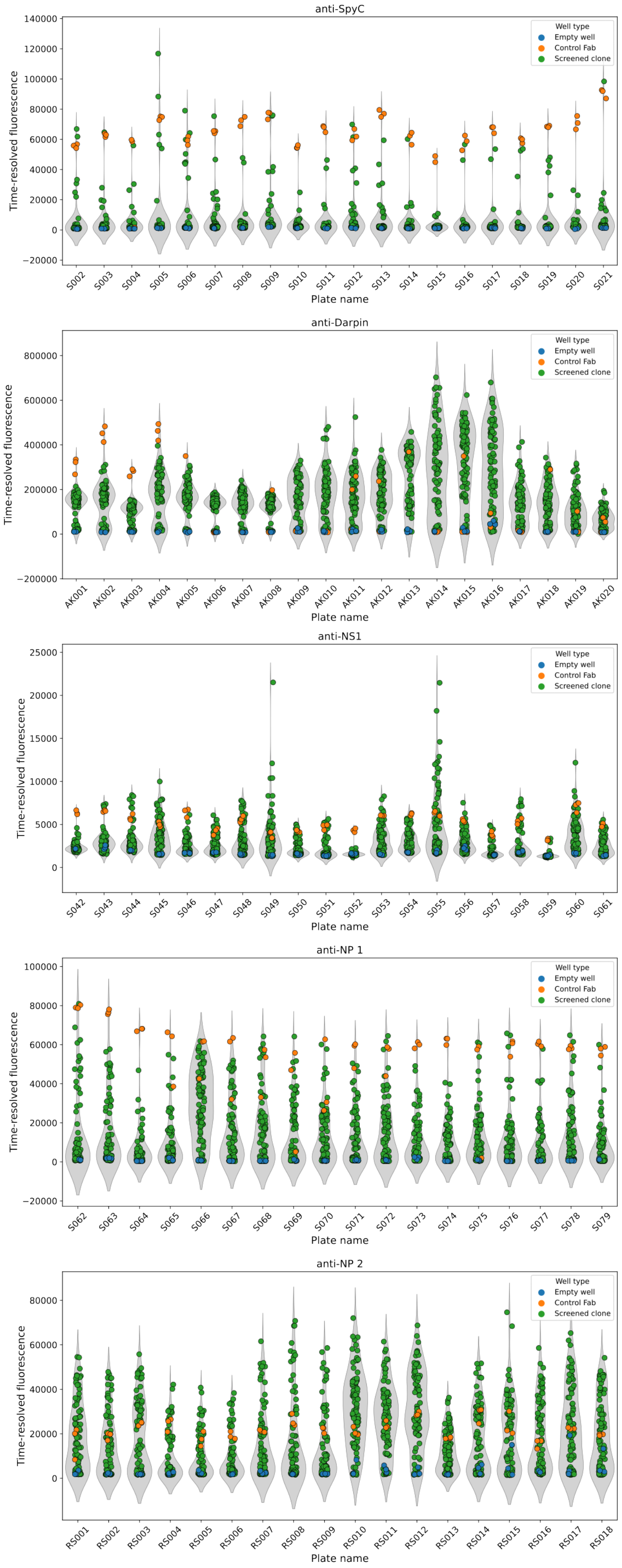

Figure S 4. Functional screening data, raw counts for each plate.

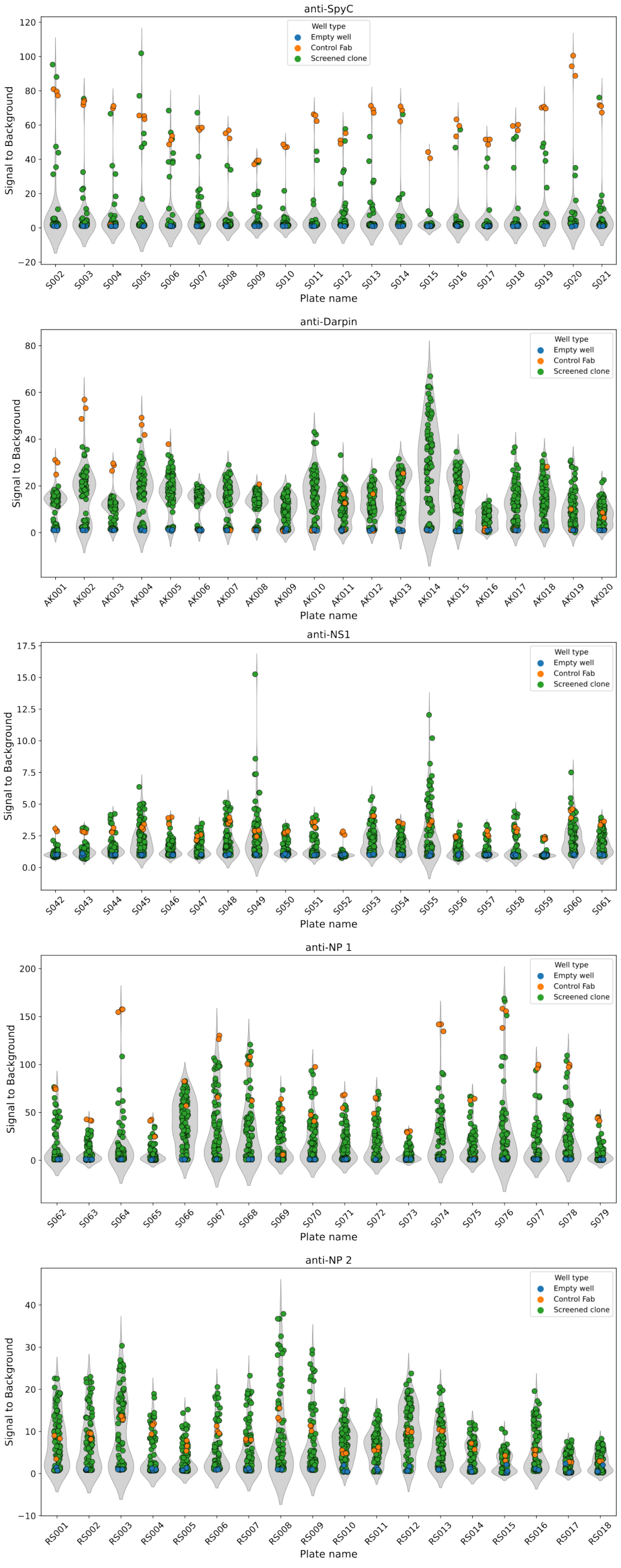

Figure S 5. Functional screening data, signal to background for each plate

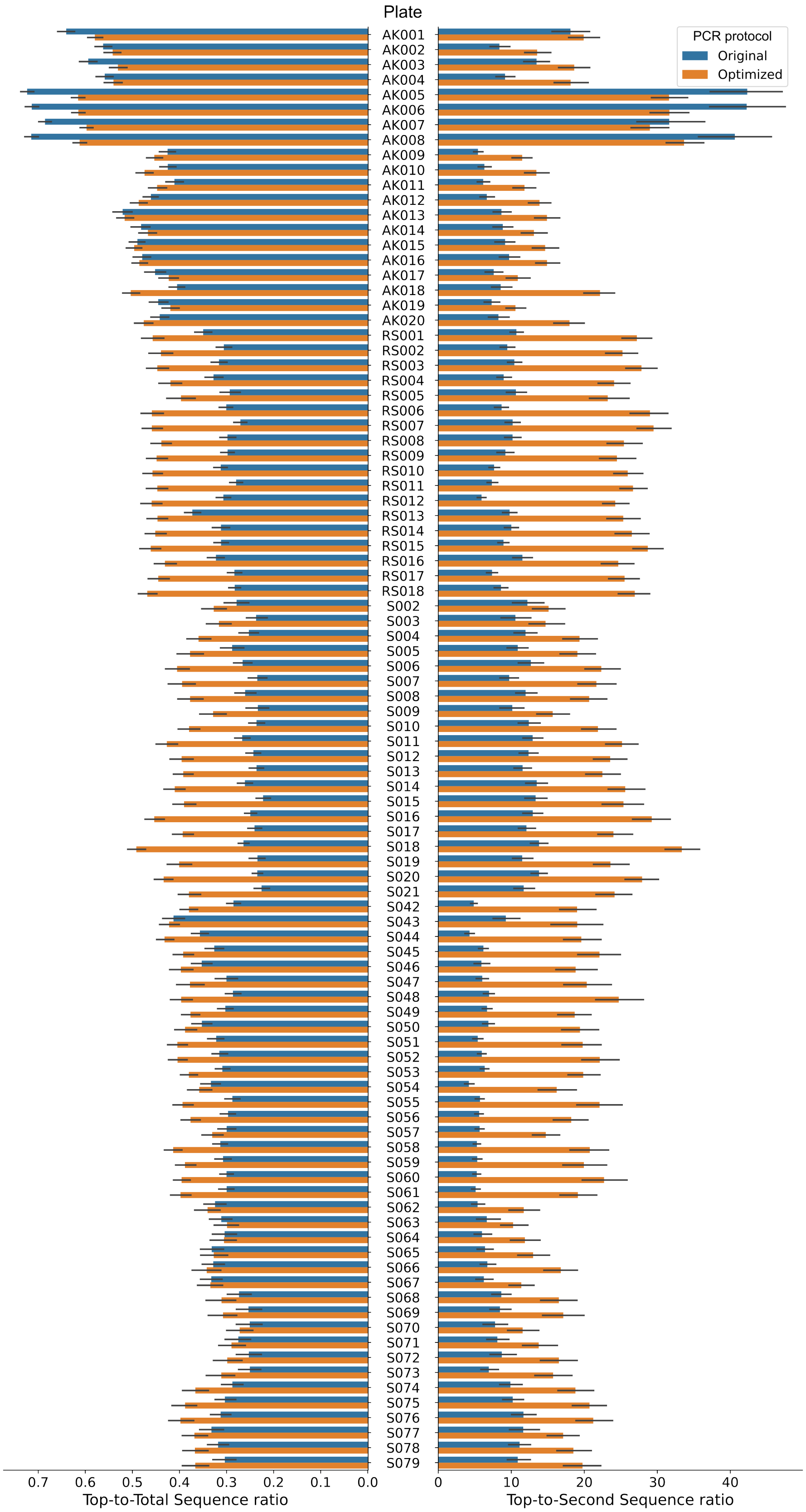

Figure S 6. Main sequence clarity improvement after optimization of plate barcoding PCR, shown as main sequence count to total sequence count (left) and main sequence count to second most abundant sequence count (right). The ratios obtained with original PCR protocol are shown in blue and with optimized in orange. Screening plate name is shown in y axis in the middle.

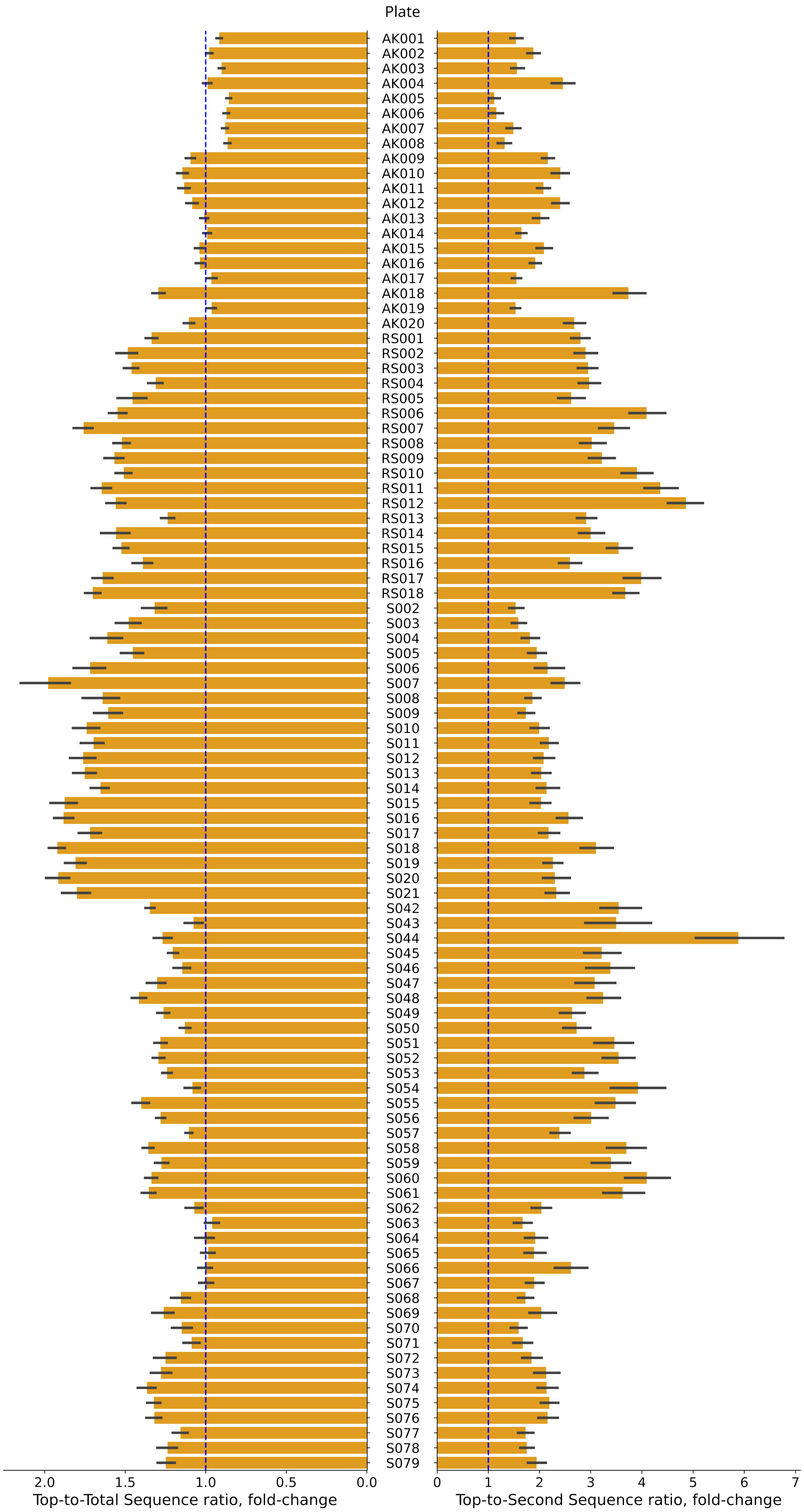

Figure S 7. Main sequence clarity improvement after optimization of plate barcoding PCR, shown as fold change of main sequence count to total sequence count (left) and main sequence count to second most abundant sequence count (right). Blue dotted line indicates fold change of 1 between original and optimized PCR protocol. Screening plate name is shown in y axis in the middle.

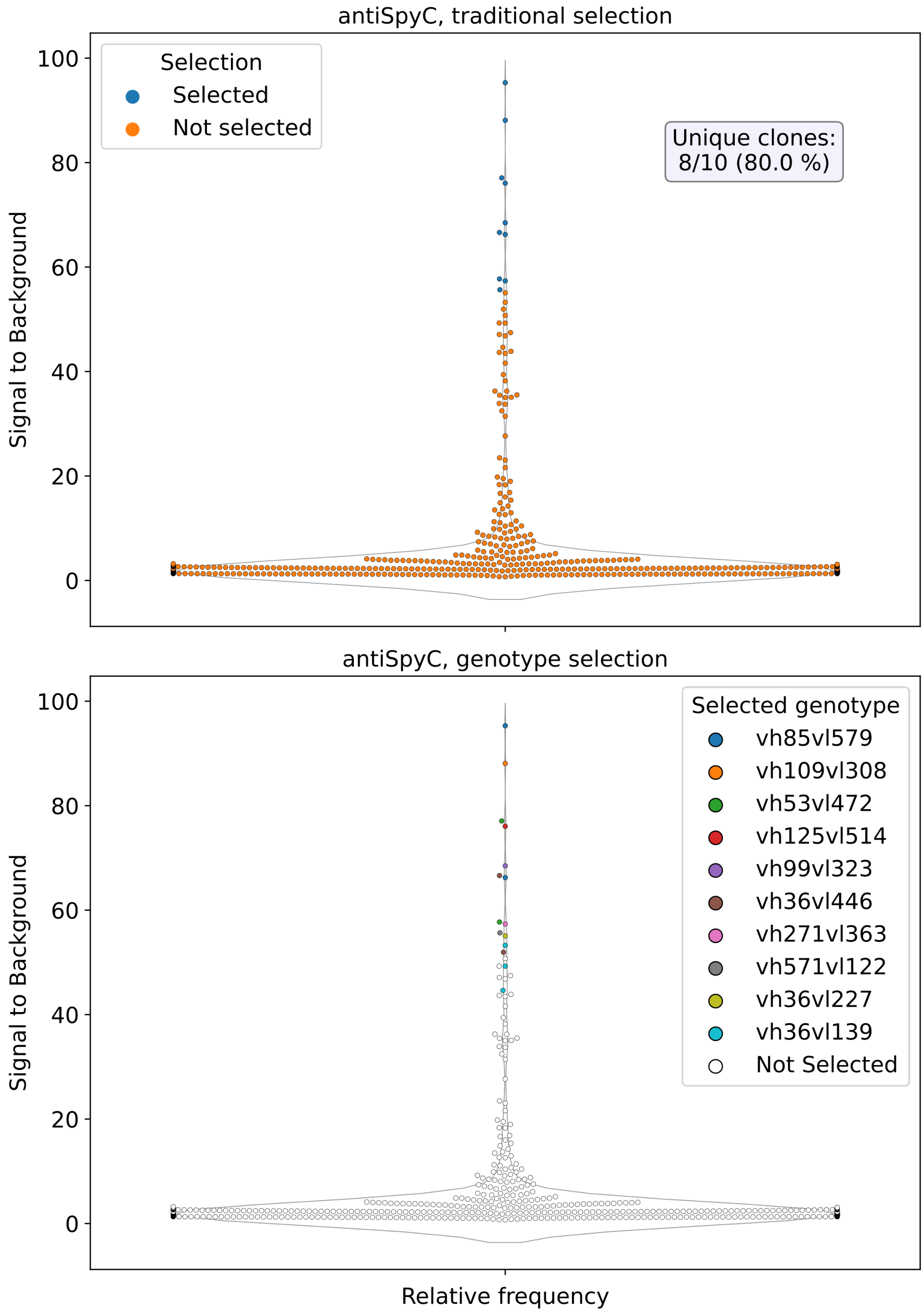

Figure S 8. Traditional (top) and genotyped (bottom) selection of 10 most promising clones of anti-SpyCatcher libraries. Traditionally selected clones are shown in orange. All clones selected based on genotype are marked coloured and non-selected with white markers. 68.2 % of the points with lowest S/B are not visible in the plot.

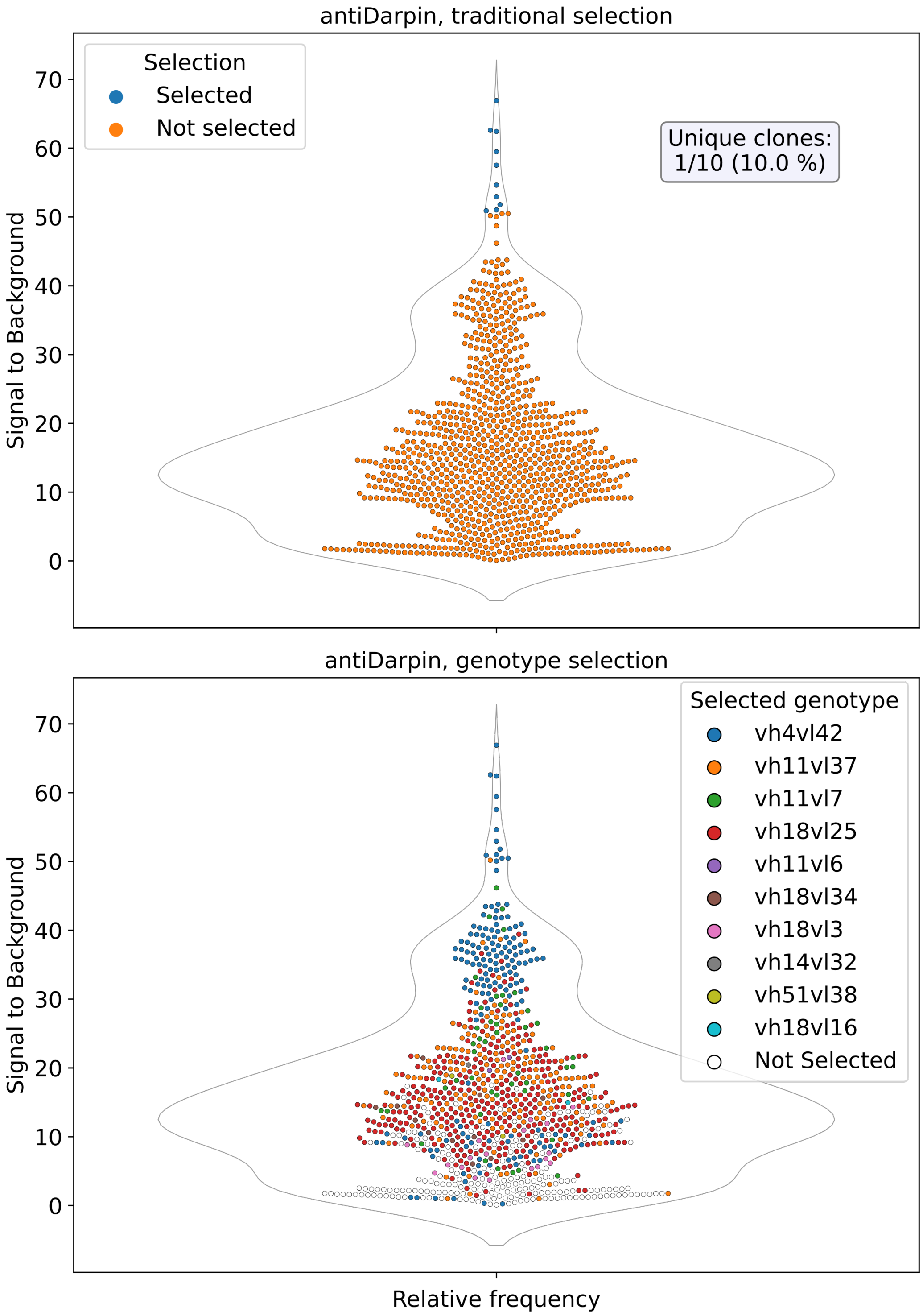

Figure S 9. Traditional (top) and genotyped (bottom) selection of 10 most promising clones of anti-DARPin library. Traditionally selected clones are shown in orange. All clones selected based on genotype are marked coloured and non-selected with white markers.

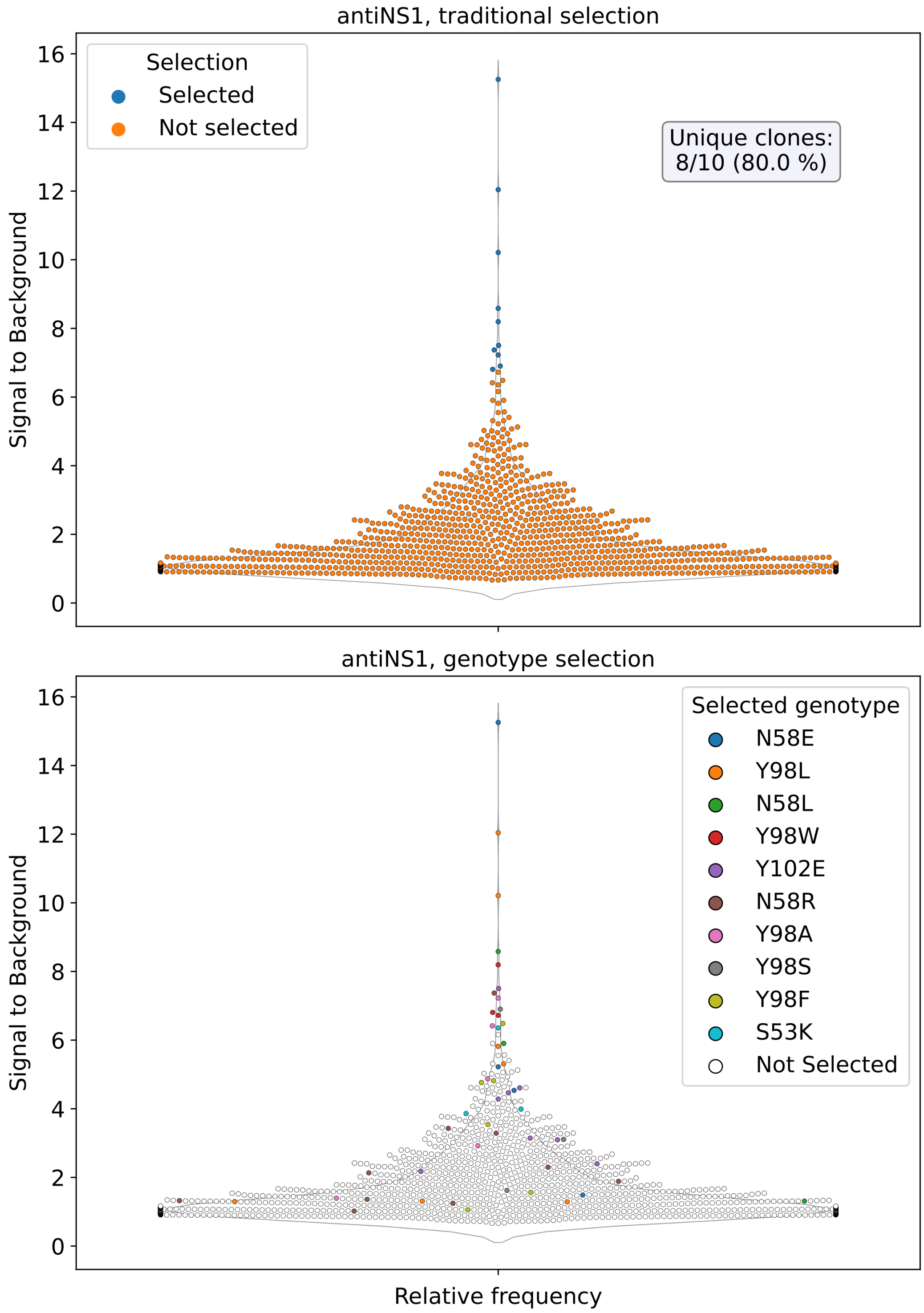

Figure S 10. Traditional (top) and genotyped (bottom) selection of 10 most promising clones of anti-NS1 library. Traditionally selected clones are shown in orange. All clones selected based on genotype are marked coloured and non-selected with white markers. 28.9 % of the points with lowest S/B are not visible in the plot.

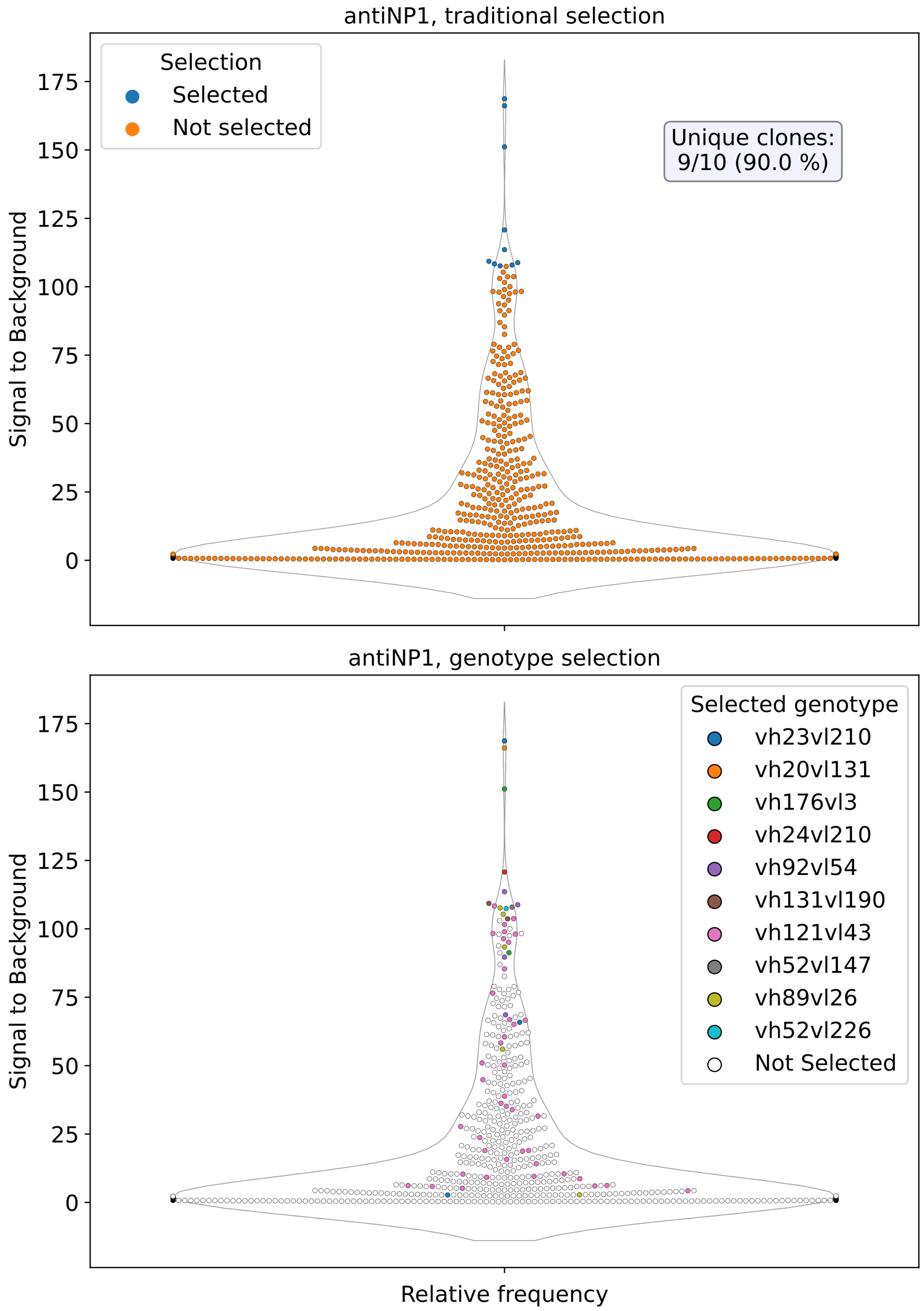

Figure S 11. Traditional (top) and genotyped (bottom) selection of 10 most promising clones of anti-NP1 library. Traditionally selected clones are shown in orange. All clones selected based on genotype are marked coloured and non-selected with white markers. 34.2 % of the points with lowest S/B are not visible in the plot.

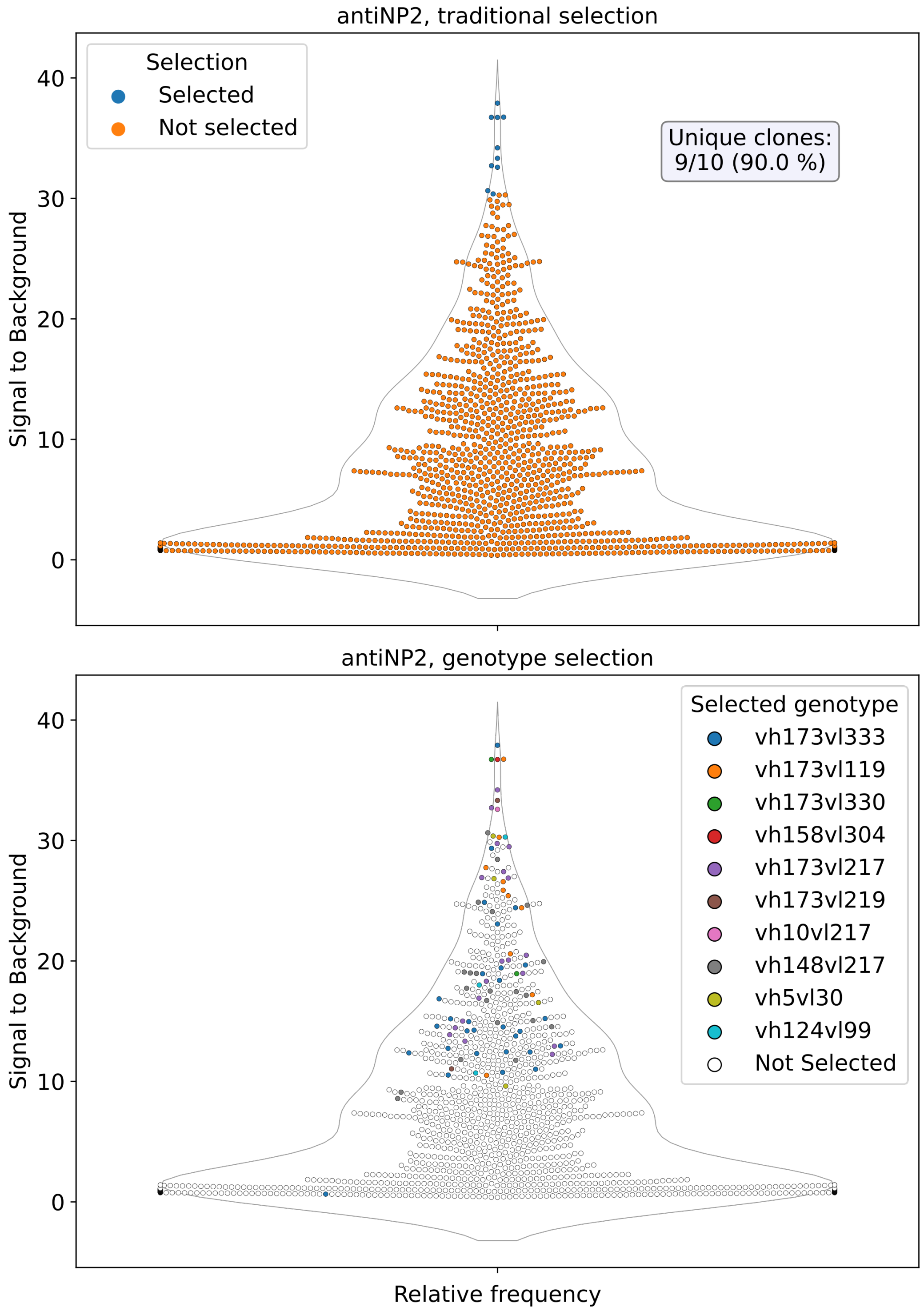

Figure S 12. Traditional (top) and genotyped (bottom) selection of 10 most promising clones of anti-NP2 library. Traditionally selected clones are shown in orange. All clones selected based on genotype are marked coloured and non-selected with white markers. 11.1 % of the points with lowest S/B are not visible in the plot.

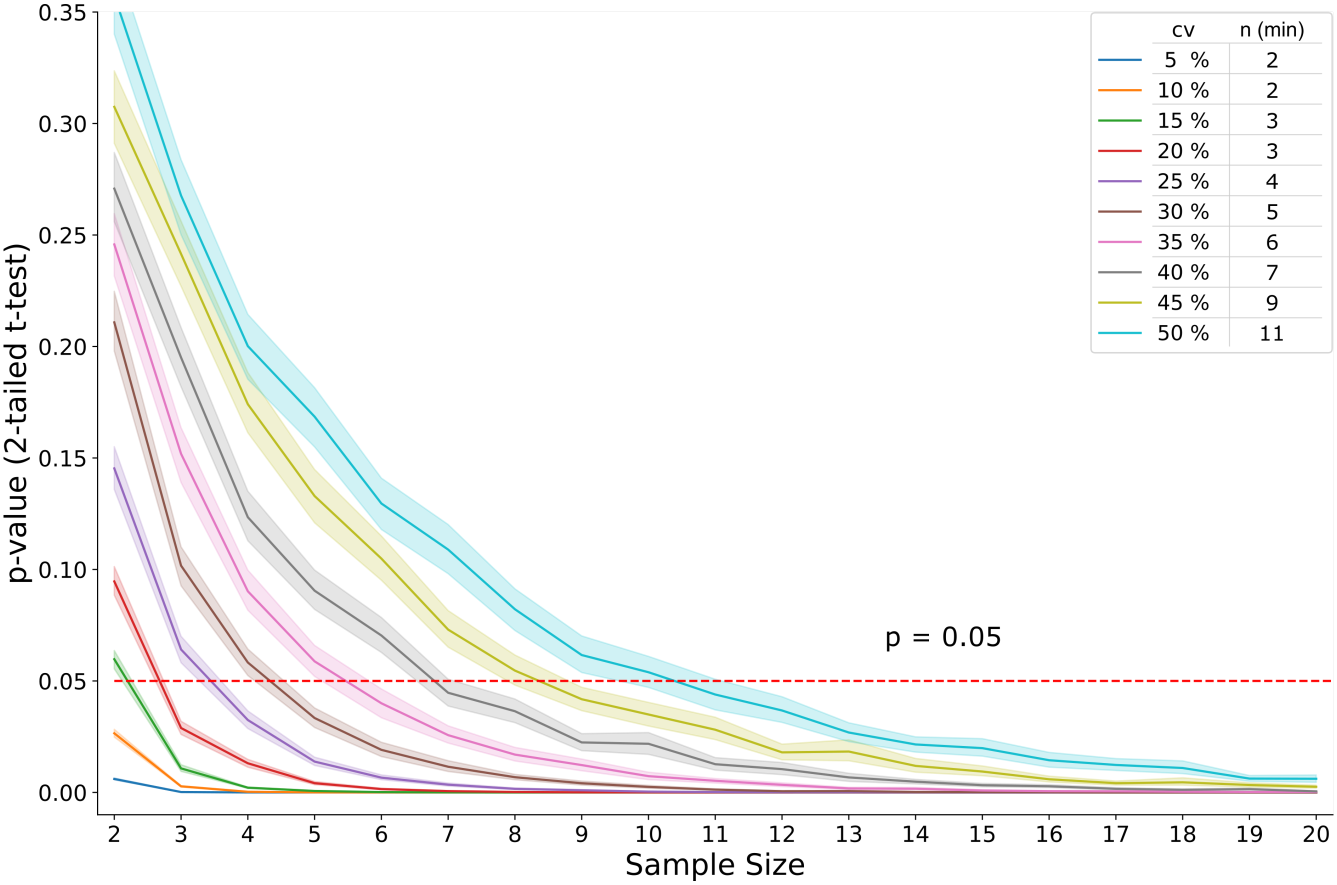

Figure S 13. Simulated two-tailed t-test results to detect two-fold change in S/B between two groups of same size and variance. Each sample size and variance combination was tested 1 000 times, each time with new normally distributed S/B values with means at three and six. Each variance level is illustrated with different colors and threshold level of statistical significance ($p = 0.05$) with red dotted line. The bands around lines indicate 95 % confidence interval. Minimum sample size to detect two-fold difference is shown in legend table. With cv $\leq$ 20 %, sample size of three is sufficient to see statistically significant difference.

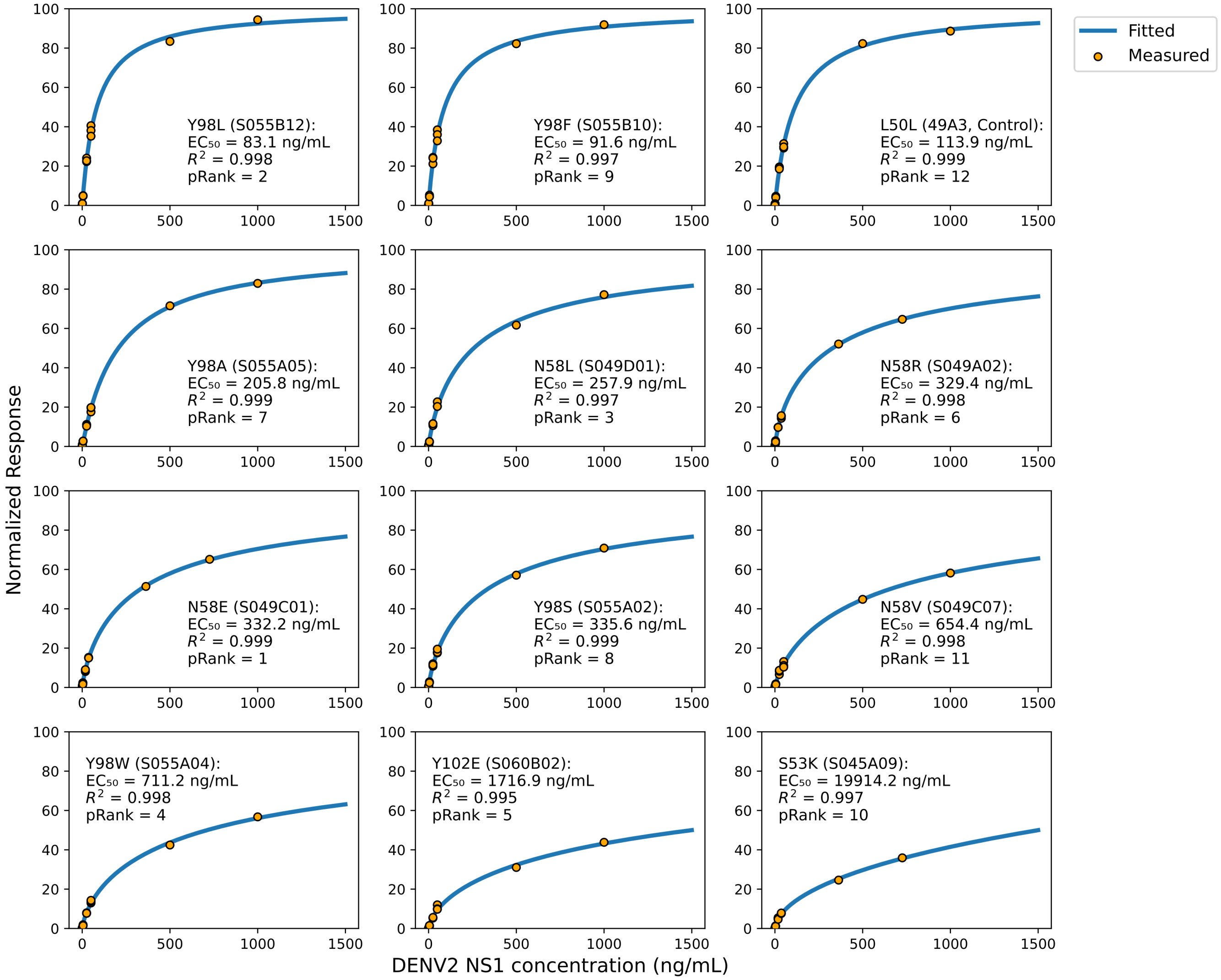

Figure S 14. Apparent affinity measurements of anti-NS1 Fab clones. Clones are ordered from left to right, top to bottom based on ascending EC_{50} values. Genotype (clone name), EC_{50}, R^2 and primary screening rank (pRank) are shown in each plot. Ranks assessed during primary screening did not correlate with the apparent affinity rank. Measured data points shown in orange and fitted four parameter log-logistic curve in blue.

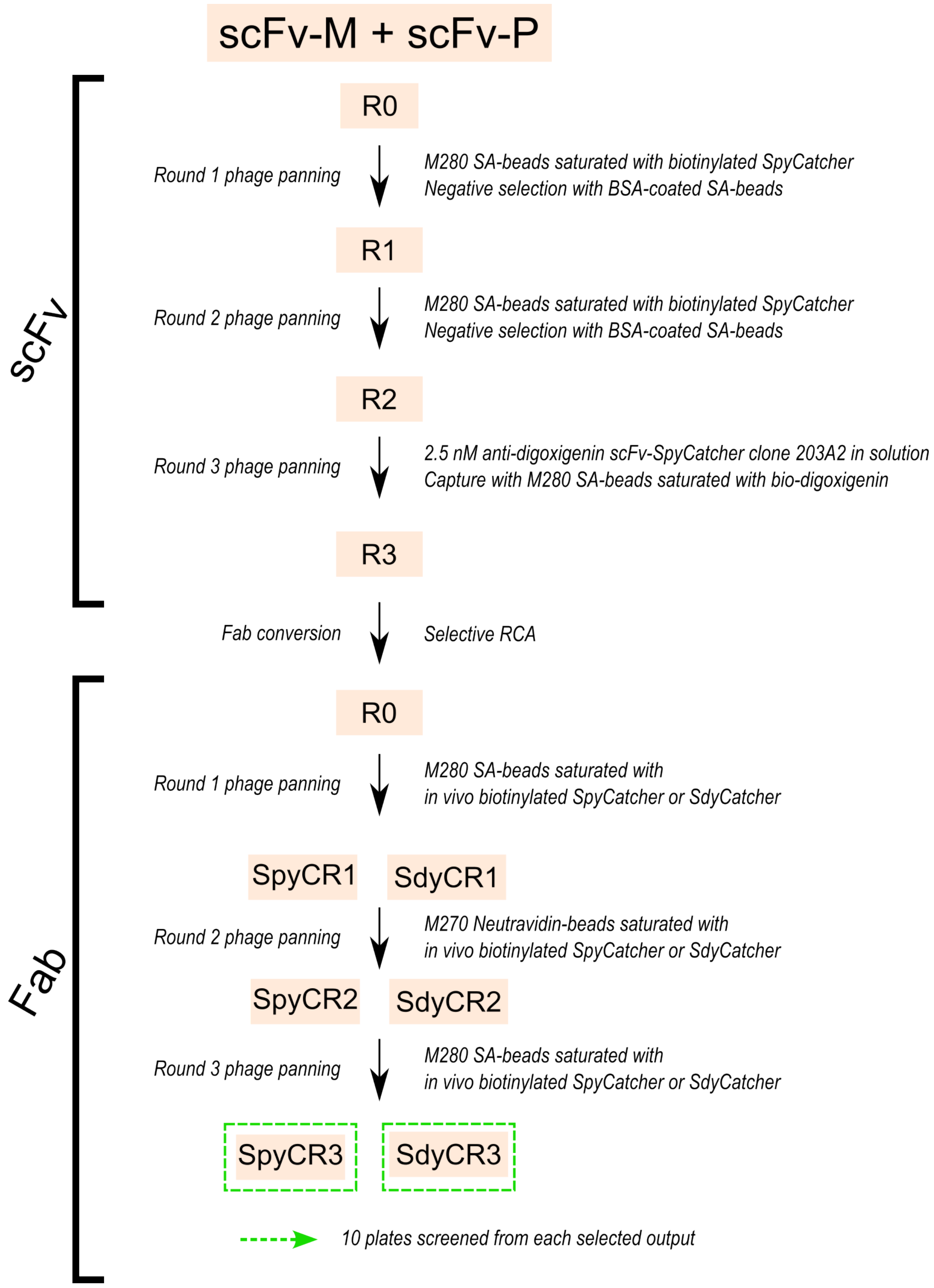

Figure S 15. Phage display selections of anti-SpyCatcher libraries for screening. Combination of scFv-M and scFv-P libraries were first panned against biotinylated SpyCatcher for two rounds, then one round against scFv-SpyCatcher, followed by conversion into Fab format via selective RCA. Fab phages were then panned against *in vivo* biotinylated SpyCatcher or SdyCatcher for three more rounds, after which the Fab genes of selected outputs (green dotted box) were cloned into pAK400 expression vector for soluble screening. "R" in the name indicates the round number of the phage library.

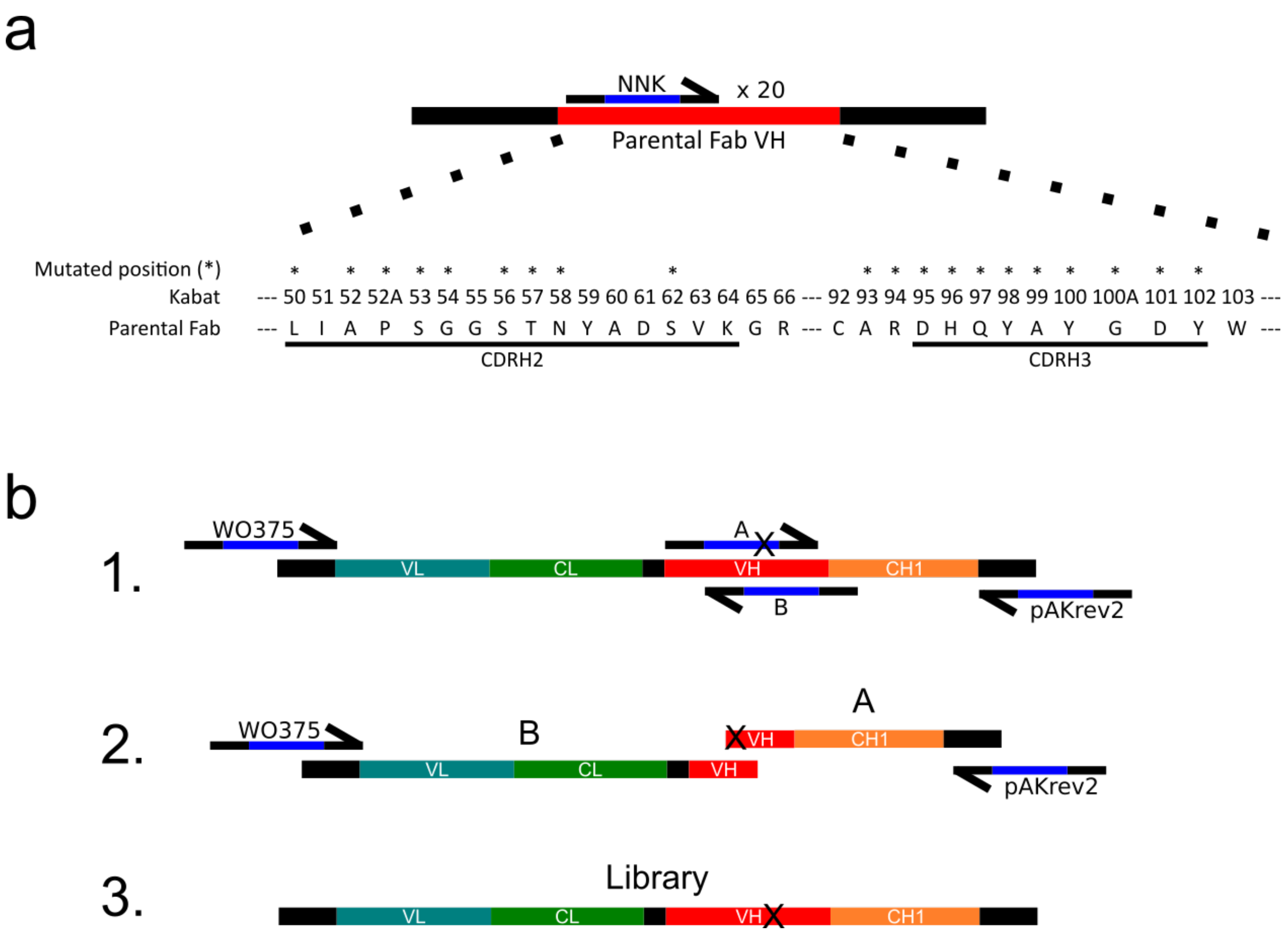

Figure S 16. Generation of anti-NS1 Fab affinity maturation library by site-saturation mutagenesis PCR. (a) Kabat numbered positions displayed on template Fab 49A3 amino acid sequence, with targeted mutation positions marked with "*". Total of 20 positions were targeted. (b) Generation of libraries using NNK-primers, mutation marked with "X". 1: Two fragments were generated with PCR for each library, mutated one with NNK-primer and pAKrev2 and non-mutated one with WO375 and primer B. 2: Assembly PCR using the two fragments generated, amplified with WO375 and pAKrev2 primers. 3: Ready Fab library, ready to be cloned into expression vector for screening.

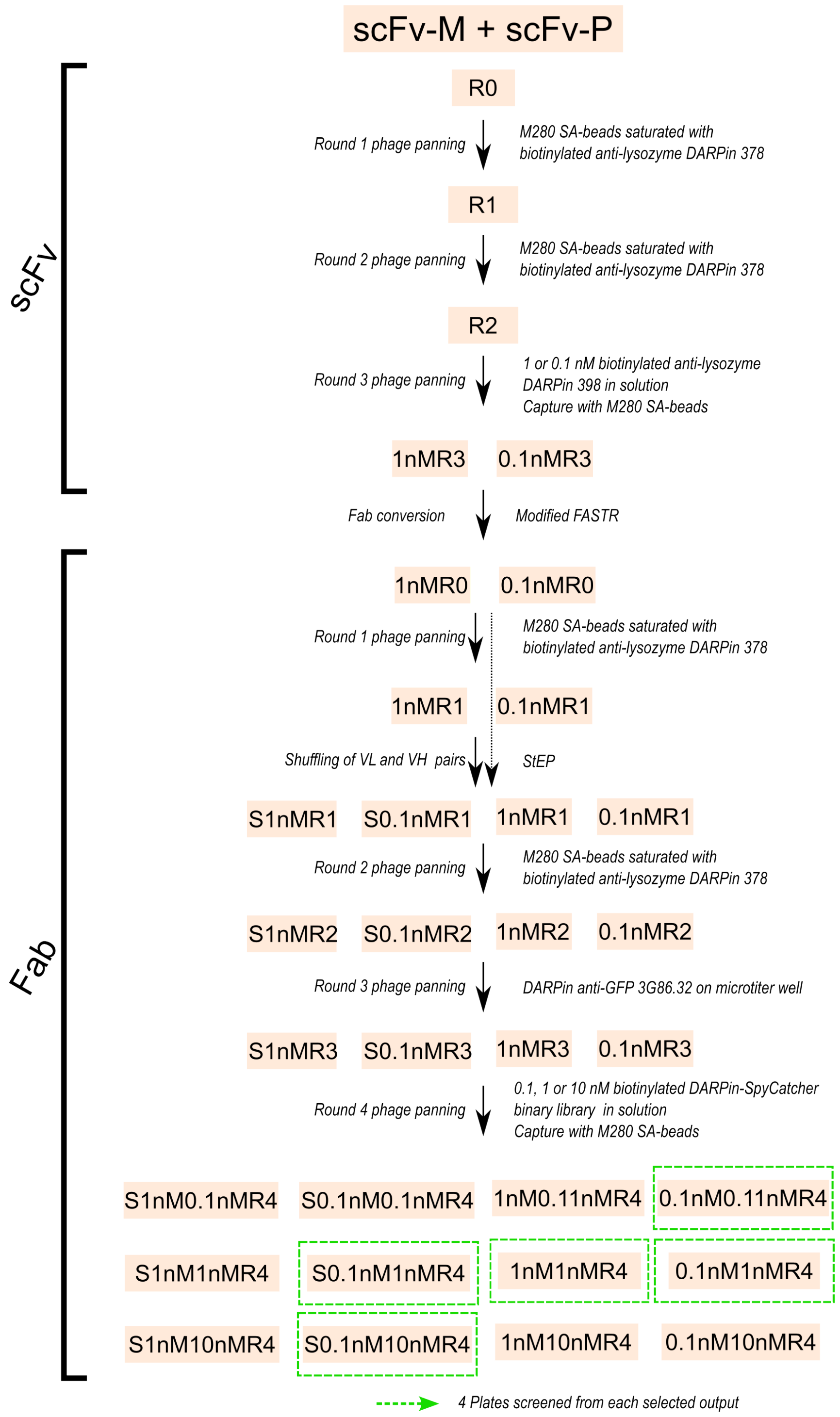

Figure S 17. Phage display selections of anti-DARPin libraries for screening. Combination of scFv-M and scFv-P libraries were first panned for two rounds against biotinylated anti-lysozyme DARPin clone 378 on SA-beads, followed by 1 round of selection against different clone in solution with 0.1 and 1 nM concentrations. The two libraries were then converted into Fab format via modified FASTR method. First round of Fab phage panning was done similarly to first round of scFv panning, followed by shuffling of VL-VH pairs via StEP. Both shuffled and non-shuffled libraries were then panned for three more rounds with antigen bound to SA-beads, microtiter well or in solution with different concentrations. The Fab genes of selected outputs (green dotted box) were cloned into pAK400 expression vector for soluble screening. "R" in the name indicates the round number of the phage library, while "S" indicates that the library underwent a shuffling step. Antigen concentrations used in solution panning are also indicated in the library output name (values ending in nM).

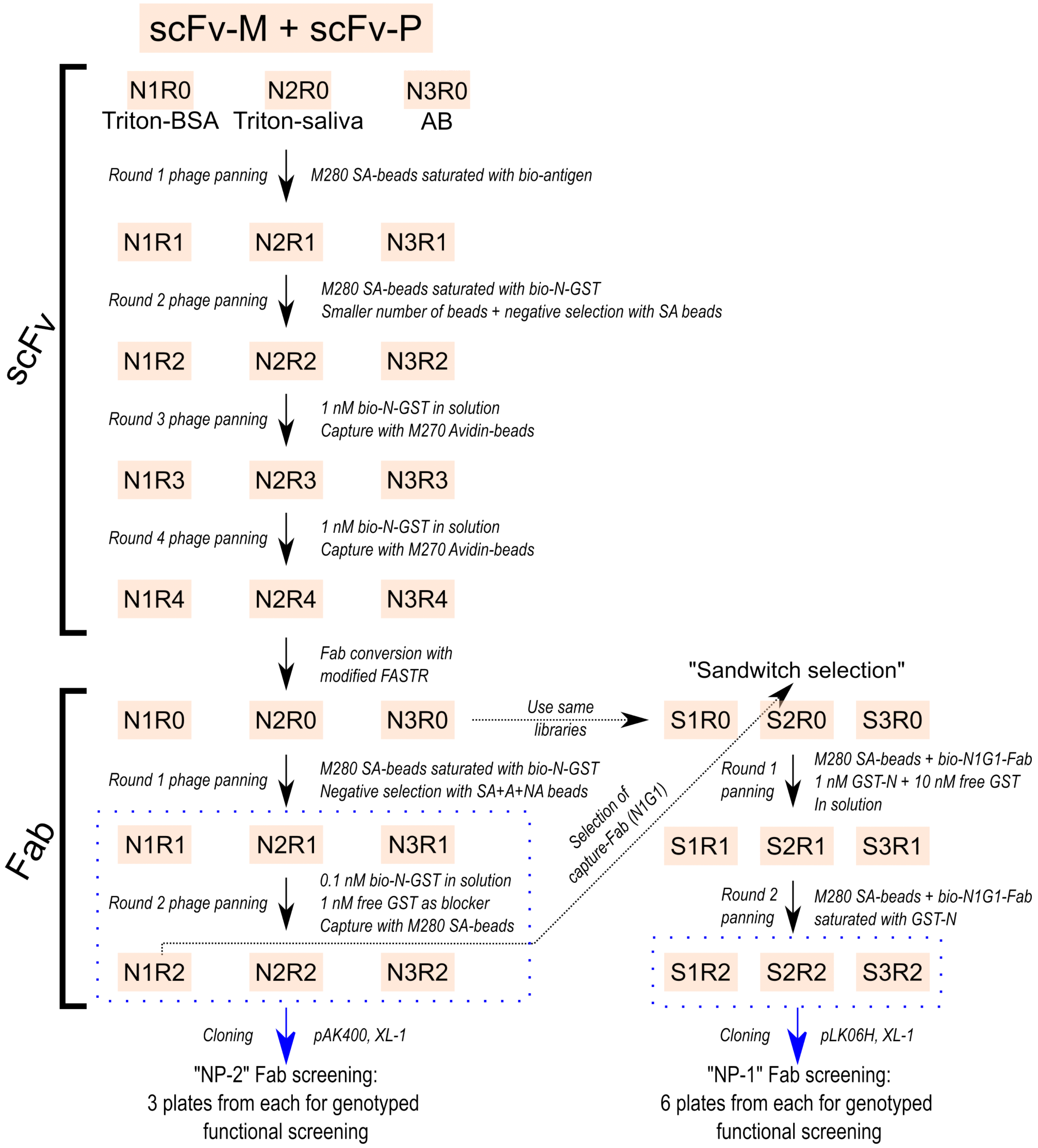

Figure S 18. Phage display selections of anti-NP libraries for screening. Three libraries were panned from combination of scFv-M and scFv-P libraries, with different additives to panning buffer; N1 with added Triton-X + BSA, N2 with added Triton-X + saliva and N3 without additives. After 4 rounds of scFv-phage selections, genes were converted into Fab format, followed with 2 more rounds of panning either similarily as in earlier rounds, or as a sandwitch with discovered N1G1 Fab bound first on magnetic beads. "R" in the name indicates the round number of the phage library.

References

1. Huovinen, T. *et al.* Two scfv antibody libraries derived from identical vl–vh framework with different binding site designs display distinct binding profiles. *Protein Eng. Des. Sel.* **26**, 683–693, DOI: 10.1093/PROTEIN/GZT037 (2013).
2. Huovinen, T. *et al.* Primer extension mutagenesis powered by selective rolling circle amplification. *PLoS ONE* **7**, e31817, DOI: 10.1371/journal.pone.0031817 (2012).
3. Kulmala, A., Huovinen, T. & Lamminmäki, U. Effect of DNA sequence of Fab fragment on yield characteristics and cell growth of E. coli. *Sci. reports* **7**, DOI: 10.1038/S41598-017-03957-6 (2017).
4. Brauchle, M. *et al.* Protein interference applications in cellular and developmental biology using darpins that recognize gfp and mcherry. *Biol. Open* **3**, 1252–1261, DOI: 10.1242/BIO.201410041 (2014).
5. Kulmala, A., Lappalainen, M., Lamminmäki, U. & Huovinen, T. Synonymous Codons and Hydrophobicity Optimization of Post-translational Signal Peptide PelB Increase Phage Display Efficiency of DARPins. *Cite This: ACS Synth. Biol* **2022**, 3181, DOI: 10.1021/acssynbio.2c00260 (2022).
6. Zhao, H., Giver, L., Shao, Z., Affholter, J. A. & Arnold, F. H. Molecular evolution by staggered extension process (StEP) in vitro recombination. *Nat. Biotechnol. 1998 16:3* **16**, 258–261, DOI: 10.1038/nbt0398-258 (1998).
7. Sanmark, H. *et al.* Fast conversion of scfv to fab antibodies using type iis restriction enzymes. *J. Immunol. Methods* **426**, 134–139, DOI: 10.1016/J.JIM.2015.08.005 (2015).
8. van den Brink, E. N. *et al.* Molecular and biological characterization of human monoclonal antibodies binding to the spike and nucleocapsid proteins of severe acute respiratory syndrome coronavirus. *J. Virol.* **79**, 1635–1644, DOI: 10.1128/JVI.79.3.1635-1644.2005/ASSET/BF91482E-ED92-49C4-808D-B111B0104E1D/ASSETS/GRAPHIC/ZJV0030557050009.JPEG (2005).

Processing of sequencing reads

This section describes the pipeline used to process the raw FASTQ files into usable, single-clone level sequences. The pipeline includes quality analysis, merging of paired-end reads, demultiplexing, trimming adapter sequences, and obtaining unique read counts and the main sequences for each index location. We also provide descriptions of the UNIX and Python scripts used in this process. Arrows at the beginning of a code row indicate that it is part of the previous line's code. The first level of demultiplexing was performed automatically by the Illumina software after sequencing, resulting in separate FASTQ files for all screening plates. For the automatic demultiplexing step, a sample sheet was provided to the instrument, which included sample names and their corresponding TruSeq indexes.

Quality analysis of FASTQ files

FASTQC v0.11.9 was utilized to assess the quality of individual FASTQ files before and after merging the paired-end reads using UNIX commands in Ubuntu 16. A list of Illumina universal and index adapter sequences was provided to the software via the 'adapter_list.txt' file. The following code was used to generate quality reports for the raw sequencing files:

```
fastqc raw_sequences/* -o quality/fastqc_raw -a tables/adapter_list.txt -t 4
```

The reports of individual FASTQ files were then combined into single report using MultiQC v1.11 with following UNIX command:

```
multiqc quality/fastqc_raw -i "SO_Fab_Seq raw FASTQ quality report" -o
 ↪ quality/multiqc_raw/ -n raw_multiqc_report
```

Merging of paired-end reads with PEAR

PEAR v0.9.11 was employed to merge the paired-end reads into complete variable domain sequences. The software was executed within a loop using a custom Bash script, which is provided below:

```
#!/bin/bash
# A script to merge read 1 and read 2 with no prior quality trimming.

# Loop through plate names from a file and perform merge:
dos2unix <../../tables/plate_names.txt | \
while read -r plate
do
        echo -ne "Processing plate $plate\r"

        fwd=../../../21-10-13_Fab_Seq_New/raw_sequences/${plate}*R1*
        rev=../../../21-10-13_Fab_Seq_New/raw_sequences/${plate}*R2*
        ./../../software/pear-0.9.11/bin/pear -f $fwd -r $rev -o
         ↪ ../../pear_merged_new/$plate -v 25 -m 600 -n 300 -q 20 -y 4G -j 6 >
         ↪ ../../pear_merged_new/$plate.log

done
```

Parameters used with PEAR were:

-f forward read (R1)

-r reverse read (R2)

-o output

-v minimum required overlap for merging, 25 bases

-m maximum read length after merging, 600 bases

-n minimum read length after merging, 300 bases

-q minimum mean quality score of 2 subsequent bases - trim sequence after quality lower than 20

-y RAM allocation, 4 Gb

-j threats used, use 6 cpu threats

After merging, the read quality was again analyzed as shown in previous subsection.

Demultiplexing of well barcodes and separation VH and VL sequences

Demultiplexing of well barcodes (individual clones) was performed using a custom Bash script within a nested loop. The first loop scanned through the merged FASTQ files of each plate, while the second loop iterated through a table that contained the well name, index 1 regular expression pattern, and index 2 regular expression pattern. These patterns were utilized with the agrep tool to identify matching sequences:

```bash
#!/bin/bash
# A script to demultiplex merged,indexed FASTQ-files, where indexes are at the ends of sequences:
# Loop through plate names:
dos2unix <../../tables/plate_names.txt | \
while read -r plate
do
        echo -ne "Processing plate ${plate}\r"
        # Loop through the well names and corresponding index-regular expressions:
        while read -r well ix1 ix2
        do
                # First split according to the indexes to separate FASTA-files per well, addition of header in FASTA format with running numbering:
                agrep -1 -e $ix1 ../../pear_merged_new/${plate}.assembled.fastq | agrep -1 -e $ix2 | awk '{print ">"NR"\n"$0}' >> ../../demultiplexed/new/wells/$plate$well.fasta

                # Second split according to the variable domains (VH/VL) to separate fasta-files --> easy matching with egrep due to single framework library

                # VH:
                egrep ../../demultiplexed/new/wells/$plate$well.fasta -e 'GAGGTA.{250,}AGCTCG' -B1 --no-group-separator >> ../../demultiplexed/new/samples/${plate}${well}_VH.fasta

                # VL (skip anti-Dengue, as only VH was sequenced):
                if [ ! $(echo $plate | egrep -e "S0[45][0-9]|S06[01]") ]
                then
                        egrep ../../demultiplexed/new/wells/$plate$well.fasta -e 'GAAATT.{250,}AAACGG' -B1 --no-group-separator >> ../../demultiplexed/new/samples/${plate}${well}_VL.fasta

                fi
        done < ../../tables/well_regex_separate.txt &

        # Run another loop in parallel, if number of jobs is less than number of CPU cores:
        [ $( jobs | wc -l ) -ge $( nproc ) ] && wait
done
```

The table "well_regex_separate.txt" containing the listed index regular expressions is provided below:

A01	^CCTAAA	TGATGG$
A02	^TGCAGA	TGATGG$
A03	^CCATCA	TGATGG$
A04	^GTGGTAT	TGATGG$
A05	^ACTTTAA	TGATGG$
A06	^GAGCAAC	TGATGG$
A07	^TGTTGCGT	TGATGG$
A08	^ATGTCCGA	TGATGG$
A09	^AGGTACGC	TGATGG$
A10	^ACAGCCACC	TGATGG$
A11	^TGTCTCGCA	TGATGG$
A12	^GAGGAGTAA	TGATGG$
B01	^CCTAAA	CGTAAC$
B02	^TGCAGA	CGTAAC$
B03	^CCATCA	CGTAAC$
B04	^GTGGTAT	CGTAAC$
B05	^ACTTTAA	CGTAAC$
B06	^GAGCAAC	CGTAAC$
B07	^TGTTGCGT	CGTAAC$
B08	^ATGTCCGA	CGTAAC$
B09	^AGGTACGC	CGTAAC$
B10	^ACAGCCACC	CGTAAC$
B11	^TGTCTCGCA	CGTAAC$
B12	^GAGGAGTAA	CGTAAC$
C01	^CCTAAA	ATACCAC$
C02	^TGCAGA	ATACCAC$
C03	^CCATCA	ATACCAC$
C04	^GTGGTAT	ATACCAC$
C05	^ACTTTAA	ATACCAC$
C06	^GAGCAAC	ATACCAC$
C07	^TGTTGCGT	ATACCAC$
C08	^ATGTCCGA	ATACCAC$
C09	^AGGTACGC	ATACCAC$
C10	^ACAGCCACC	ATACCAC$
C11	^TGTCTCGCA	ATACCAC$
C12	^GAGGAGTAA	ATACCAC$
D01	^CCTAAA	TTAAAGT$
D02	^TGCAGA	TTAAAGT$
D03	^CCATCA	TTAAAGT$
D04	^GTGGTAT	TTAAAGT$
D05	^ACTTTAA	TTAAAGT$
D06	^GAGCAAC	TTAAAGT$
D07	^TGTTGCGT	TTAAAGT$
D08	^ATGTCCGA	TTAAAGT$
D09	^AGGTACGC	TTAAAGT$
D10	^ACAGCCACC	TTAAAGT$
D11	^TGTCTCGCA	TTAAAGT$
D12	^GAGGAGTAA	TTAAAGT$
E01	^CCTAAA	ACGCAACA$
E02	^TGCAGA	ACGCAACA$
E03	^CCATCA	ACGCAACA$
E04	^GTGGTAT	ACGCAACA$
E05	^ACTTTAA	ACGCAACA$
E06	^GAGCAAC	ACGCAACA$
E07	^TGTTGCGT	ACGCAACA$
E08	^ATGTCCGA	ACGCAACA$
E09	^AGGTACGC	ACGCAACA$
E10	^ACAGCCACC	ACGCAACA$
E11	^TGTCTCGCA	ACGCAACA$
E12	^GAGGAGTAA	ACGCAACA$
F01	^CCTAAA	TCGGACAT$
F02	^TGCAGA	TCGGACAT$
F03	^CCATCA	TCGGACAT$
F04	^GTGGTAT	TCGGACAT$
F05	^ACTTTAA	TCGGACAT$
F06	^GAGCAAC	TCGGACAT$
F07	^TGTTGCGT	TCGGACAT$
F08	^ATGTCCGA	TCGGACAT$
F09	^AGGTACGC	TCGGACAT$
F10	^ACAGCCACC	TCGGACAT$
F11	^TGTCTCGCA	TCGGACAT$
F12	^GAGGAGTAA	TCGGACAT$
G01	^CCTAAA	GGTGGCTGT$
G02	^TGCAGA	GGTGGCTGT$
G03	^CCATCA	GGTGGCTGT$
G04	^GTGGTAT	GGTGGCTGT$
G05	^ACTTTAA	GGTGGCTGT$
G06	^GAGCAAC	GGTGGCTGT$
G07	^TGTTGCGT	GGTGGCTGT$
G08	^ATGTCCGA	GGTGGCTGT$

G09	^AGGTACGC	GGTGGCTGT$
G10	^ACAGCCACC	GGTGGCTGT$
G11	^TGTCTCGCA	GGTGGCTGT$
G12	^GAGGAGTAA	GGTGGCTGT$
H01	^CCTAAA	TGCGAGACA$
H02	^TGCAGA	TGCGAGACA$
H03	^CCATCA	TGCGAGACA$
H04	^GTGGTAT	TGCGAGACA$
H05	^ACTTTAA	TGCGAGACA$
H06	^GAGCAAC	TGCGAGACA$
H07	^TGTTGCGT	TGCGAGACA$
H08	^ATGTCCGA	TGCGAGACA$
H09	^AGGTACGC	TGCGAGACA$
H10	^ACAGCCACC	TGCGAGACA$
H11	^TGTCTCGCA	TGCGAGACA$
H12	^GAGGAGTAA	TGCGAGACA$

Trimming of variable domain sequences and calculating unique read counts

Sequences flanking the variable domains were removed with egrep using regular expression patterns matching for either VH or VL. Due to use of single-framework library, only one regex pattern was needed for each variable domains. After the trimming, the unique VH / VL read counts per clone were calculated using fastx_collapser tool from fastx_toolkit. Both of these steps were done using bash script below:

```
#!/bin/bash
# Remove sequences flanking VL or VH sequences, including the indexes and collapse:

### Trim ###
## VH:
ls ../../demultiplexed/new/samples/ | grep -e 'VH' | while read -r file
do
        egrep ../../demultiplexed/new/samples/$file -e 'GAGGTA.{250,}AGCTCG' -o |
         ↪  awk '{print ">"NR"\n"$0}' >> ../../demux_trimmed/new/$file
done

## VL:
ls ../../demultiplexed/new/samples/ | grep -e 'VL' | while read -r file
do
        egrep ../../demultiplexed/new/samples/$file -e 'GAAATT.{250,}AAACGG' -o |
         ↪  awk '{print ">"NR"\n"$0}' >> ../../demux_trimmed/new/$file
done

### Collapse ###
# New
ls ../../demux_trimmed/new/ | while read -r file
do
        ./../../software/fastx_toolkit/bin/fastx_collapser -i
         ↪  ../../demux_trimmed/new/$file >>
         ↪  ../../demux_trimmed_collapsed/new/$file
done
```

Sequence counts and the most abundant sequence

Total sequence count and the counts of the most and second most abundant sequences were collected separately for VH and VL in all clones. Additionally, the most abundant sequence was extracted using the following Bash script:

```
#!/bin/bash
# Bash code to count full sequence counts from each multiplexed sample fasta-file:

# Create csv for counts:

echo "Plate,Well,Chain,Type,Total_Count,Top_Count,Second_Count,Top_Sequence" >
 ↪  ../../tables/21-11-05_Full_Sequence_Counts_demux_trimmed.csv

# Loop through file names:
ls ../../demux_trimmed/new/ | while read -r file
do
        plate=$(echo $file | grep -oP -e '^[ASR]+[KS]*[0-9]{3}')
        well=$(echo $file | grep -oP -e "${plate}\K[A-H][0-9]{2}")
        chain=$(echo $file | grep -oP -e "${plate}${well}[_]\KV[LH]")

        echo -ne "Now processing sample $plate$well$chain\r"

        # New:
        total=$(grep ../../demux_trimmed/new/$file -e "^>" -c)
        top=$(head ../../demux_trimmed_collapsed/new/$file | grep -Po -e
         ↪  '>1-\K.*$')
        top_seq=$(head ../../demux_trimmed_collapsed/new/$file -n4 | sed -n '2p')
        second=$(head ../../demux_trimmed_collapsed/new/$file | grep -Po -e
         ↪  '>2-\K.*$')
        echo "$plate,$well,$chain,New,$total,$top,$second,$top_seq"  >>
         ↪  ../../tables/21-11-05_Full_Sequence_Counts_demux_trimmed.csv

done &
# Run another loop in parallel, if number of jobs is less than number of CPU cores:
[ $( jobs | wc -l ) -ge $( nproc ) ] && wait
```
